# A systematic review of the methods used to analyze the economic impact of endemic foot‐and‐mouth disease

**DOI:** 10.1111/tbed.14564

**Published:** 2022-06-20

**Authors:** Polly Compston, Georgina Limon, Barbara Häsler

**Affiliations:** ^1^ Veterinary Epidemiology, Economics and Public Health Group, Department of Pathobiology and Population Sciences Royal Veterinary College London UK; ^2^ The Pirbright Institute Pirbright UK

**Keywords:** economic methods, endemic disease, foot‐and‐mouth disease, livestock, vaccination

## Abstract

Foot‐and‐mouth disease (FMD) has large economic consequences in livestock systems, which must be robustly assessed to support disease control policy. This study described and assessed methods used within economic analyses of FMD and its control in endemic contexts. A systematic literature search was conducted in six academic search engines. Studies were included if they applied an economic analysis to a context with endemic FMD, producing a result articulated as a monetary figure. Data collected from each article included country of study, animal population, geographical level of analysis, time horizon and type of economic analysis. Each study was scored using a quality assessment tool containing a checklist of 42 reporting criteria. Sixty‐four articles were included, from 12,087 identified in the searches, describing results for 26 countries. Over half of the articles (56%) described economic impact of FMD retrospectively, often only accounting for a selection of direct costs at farm or household level. Median quality score calculated was 41% (range 8%–86%). Methods were generally poorly reported, confirming previously described difficulties in using published data to evaluate economic impact of endemic FMD. Few studies included disaggregation of public and private costs, or benefits, of FMD control, or accounted for economic or social influences of scale in vaccination programmes. Many of the studies included had gaps in both premise and methodology. If these analyses are used when planning and budgeting FMD control programmes in endemic contexts, there is a risk of inefficient resource allocation.

## 1 INTRODUCTION

Foot‐and‐mouth disease (FMD) is commonly cited as one of the most important livestock diseases because of its large economic consequences. These consequences are different between contexts where the disease is endemic or introduced into an FMD‐free area (epidemic), and have been described in previous literature reviews (Knight‐Jones & Rushton, 2013; Knight‐Jones et al., 2017). Knight‐Jones and Rushton (2013) identified impacts of FMD globally using literature review and expert opinion, and categorized these impacts into direct and indirect losses using a previously‐developed framework (Rushton, 2012). Subsequently, they discussed the impact of FMD in countries depending on their disease status and trade prospects. They developed a model that consolidated data from contexts where FMD is endemic, resulting in an annual estimate of costs caused by production losses and vaccination between 6.5 billion and 21 billion USD. Knight‐Jones et al. (2017) built on this analysis to focus on smallholders, defined in their paper as ‘economically vulnerable households whose income significantly depends upon FMD‐susceptible livestock (principally cattle, water buffalo, goats, sheep and pigs) [and that includes] pastoralists and agro‐pastoralists’. This review identified a fragmented body of literature, outlined key gaps in knowledge (which included geographical distributions, production systems, species and epidemiology) and proposed a framework for future economic analyses of FMD in smallholder systems, including discussion of appropriate study designs and characteristics, to improve comparability between future studies. However, there has not been a systematic review of the methods used to analyze the economics of endemic FMD and its control.

Endemic disease has been defined as ‘disease which is most of the time, and therefore ‘‘normally’’, present in a population’ (Pfeiffer, 2010). The definition of ‘normal’ varies by location and can include a wide range of incidence and prevalence estimates, depending on what is agreed to be acceptable. FMD is one of six animal diseases for which the World Organisation for Animal Health (OIE) officially recognizes disease‐free status for trade purposes. The Progressive Control Pathway for Foot and Mouth Disease (PCP‐FMD) describes a series of structured steps through which countries advance in order to obtain increasing FMD control, ultimately gaining OIE recognition of freedom from disease (FAO et al., 2018). This creates a *de facto* definition of endemic FMD for any areas where freedom from disease is not established. The PCP‐FMD is integral to the Global Foot‐and‐mouth Control Strategy (OIE & FAO, 2012), which identifies in its first paragraph the economic disparity experienced between areas where freedom from disease has been established and those where FMD is endemic. Most countries with endemic FMD are categorized as low or middle income by the World Bank, with large populations of smallholder and subsistence farmers in both sedentary and pastoralist livestock systems. In addition, there are likely to be other considerations that impact economic analysis, for example, availability of monitoring and surveillance data, concurrent endemicity of other important livestock diseases and different priorities for policy and other decision‐makers within the livestock system.

Many different frameworks, and methods, are used to evaluate the economic impact of animal disease and economic efficiency of disease control (Rushton, 2012). Traditionally, these are categorized according to their level of analysis, at farm or household, sectoral or national levels (Stott & Gunn, 2017). Farm, or household, economic studies will generally use methods that model production impact on enterprise budgets, for example, partial budget analysis, often including epidemiological modelling and potentially including different disease control scenarios (e.g., Waret‐Szkuta et al., 2017). Research evaluating the consequences of disease and the value of its control on a sector may include cost‐benefit and cost‐effectiveness analyses, as well as supply and demand and their influence on price, and therefore market dynamics (e.g., Aragrande & Canali, 2017). Studies evaluating how disease influences larger geographical administrative regions, for example, national level analysis, can include calculation of public animal health programme costs and extended economic impacts on disparate industries as well as epidemiology (e.g., Hayama et al., 2017), potentially bringing wider considerations by using system dynamic modelling and partial equilibrium models. Further delineation can be achieved through considerations of endemic or epidemic disease (Stott & Gunn, 2017), with increasing integration of behavioural economics (Garza et al., 2020) and social science (Bennett, 2017).

The economic consequences of animal health differ across species, production systems, geographical region and reasons for animal ownership. These divergent contexts interact with methods used for economic analysis: an example is demonstrated in Peck and Bruce (2017), who compare brucellosis control in Albania and the United States, demonstrating the importance of considering these contextual differences in disease control programme evaluation. Information about which methods are most commonly used and applied to specific contexts, and their respective strengths and limitations, can be used to inform future analyses. This information could then be used to populate a framework describing the minimum methodological requirements for different combinations of production system and research question. If different methods are used to evaluate similar situations, it can be difficult to compare or interpret results.

Previous systematic reviews evaluating the economic impact of endemic animal disease have used varying types and degree of quality assessment. For example, in a review of the economic evidence regarding tuberculosis in cattle, Caminiti et al. (2016) used a 35‐question checklist developed for human healthcare analyses (La Torre et al., 2011). Pinior et al. (2017) used a modified version of the 19‐question tool described by Evers et al. (2005) in their review of bovine viral diarrhoeal virus. These tools have been succeeded by the Consolidated Health Economic Evaluation Reporting Standards (CHEERS) statement (Husereau et al., 2013), which represents a standardized quality assessment matrix for human disease. There is no equivalent protocol available for animal health. However, the CHEERS statement may not be wholly transferable to animal health contexts. Human health economics has a uni‐faceted concern with the reduction of health burdens, compared with the multifaceted economic consequences of reduced production that arise from animal disease, which include commercial inefficiency, animal welfare, food security and ecological impacts. This influences the structure of animal health systems and policy in comparison to human healthcare. The economics of human health are more standardized, and the diversity of production systems seen in animal health is not present. Additionally, human health economics have a longer history of integration into medical practice than seen in animal healthcare.

This systematic review aimed to identify methods used to evaluate the economic impact of FMD and its control in endemic contexts. Our objectives were to (i) describe these methods, (ii) assess how these methods have been reported and (iii) assess their quality using a standardized protocol. We intended to identify economic analysis methods that are robust and result in the most applicable measures of FMD impact and efficiency of its control in endemic contexts. Additionally, it was hoped that these findings would have relevance for the economic evaluation of other livestock diseases.

## 2 METHODS

### 2.1 Search strategy

The World Health Organization's guidelines for rapid reviews (Tricco et al., 2017) were used for this literature review. They follow an outline similar to the Preferred Reporting Items for Systematic Reviews and Meta‐Analyses (PRISMA) guidelines (Moher et al., 2009), with the main adaptation being that a sole reviewer is responsible for screening and assessing the articles identified during the electronic search, and limited risk‐of‐bias analysis is performed. These guidelines were selected due to their focus on decision‐making in policy and because resources were not available for a full systematic review. A literature search was conducted and downloaded into Excel, the results combined, duplicates deleted, and the resulting list screened for relevance as described below.

A publication search was performed. PubMed, Scopus, CAB Abstracts, Science Direct, Web of Science and Google Scholar were selected to obtain a full range of papers and searched on 18th August 2020, using the following criteria:

(“Foot and mouth” OR FMD*)

AND

(economic* OR financ* OR livelihood* OR socioeconomic* OR “food security” OR impact* OR cost* OR benefit* OR loss* OR expenditure OR effectiveness OR “gross margin” OR “partial budget” OR CBA)

NOT

(“hand foot and mouth” or enterovirus* or HFMD* or “Fibromuscular Dysplasia” OR “Flow mediated dilatation” OR “frontometaphyseal dysplasia” OR “flow‐mediated dilation” OR “frequent mental distress”)

AND

yr: [1980 TO 2020].

Due to the large number of records that were retrieved from the Google Scholar search only the first 100 results were included, to capture as many relevant articles as possible whilst balancing the resource intensive extraction of search results. Only articles written in English were included. For all other search engines, all results were included.

Articles were excluded at title stage if the title indicated that they:

‐ were about a human disease syndrome, or otherwise clearly would not contain information about FMD.
‐ were laboratory based, reporting virological or immunological results.
‐ were based in a country classified as FMD‐free by the OIE.

Articles were excluded at abstract stage if they fulfilled any of the criteria above where this was not clear from the title or they:

‐ contained epidemiological data on FMD with no indication of economic analysis.
‐ described FMD control programmes with no indication of economic analysis.

Where an abstract or full text was not available electronically and the title or abstract, respectively, did not directly refer to any type of socio‐economic analysis of endemic FMD, the article was excluded. Review articles were identified, and their reference section examined for articles that had not been identified within the search. These were subjected to the same screening stages and exclusion criteria as described above.

Endemic was defined as a context that had not recently had an official OIE disease‐free status for FMD, and where outbreaks were occurring on a regular basis without introduction from outside that context. ‘Recent’ disease‐free status was identified where the aim of disease control centred on return to freedom‐from‐disease, or the impact of an FMD epidemic into an area that had had freedom‐from‐disease status.

Subsequently, full articles were read, and studies were included if they applied a quantitative economic analysis or framework to a context where FMD was endemic.

### 2.2 Data extraction

Data collected from each article included the country within which the study was set, stated objective of economic analysis, description of economic methods used, details of data collection methods, animal population included, geographical level of analysis and time horizon. Additional information deemed relevant, for example if epidemiological information was presented alongside economic results, or if more than one disease was included in analysis, was also recorded.

### 2.3 Quality assessment

Papers were assessed using a 42‐parameter framework to appraise the quality of economic analyses, which was adapted from Husereau et al. (2013) (Table 1). Scores produced were analyzed using descriptive statistics. Denominator values were evaluated for range and influence over final scores. Scoring for all papers was performed by PC.

**TABLE 1:** Framework used for appraising the quality of economic analyses in animal health, adapted from Husereau et al. (2013), with inclusion of quality parameters for articles included in the systematic review. Each quality parameter represents one point, and had to be completely fulfilled for that point to be obtained. If one requirement was not relevant (e.g., explanation of why a discount rate was chosen when use of a discount rate was unnecessary), that point was deducted from the denominator.

			Percentage of articles where parameter is:	
Section of paper	Component (maximum available score)	Quality parameter	Included in article	Not relevant
Title	Title (1)	Identify the study as an economic analysis^a^	81	0
Introduction	Context (1)	Provide an explicit statement of the broader context for the study^a^	90	0
	Research question (2)	Present the study question^a^	84	0
		Present its relevance for policy or practice decisions	51	0
Methods	Target population and subgroups (2)	Describe characteristics of the population and subgroups analyzed	65	0
		Describe why they were chosen	55	2
	Setting and location (1)	State relevant aspects of the systems for which the analysis is performed (e.g., public, private)	52	0
	Economic analysis (2)	State what method of economic analysis has been performed	63	0
		State why it was chosen	38	0
	Comparators (2)	Describe the interventions or strategies being compared^a^, ^c^	96	60
		State why they were chosen^a^, ^c^	88	60
	Time horizon (1)	State the time horizon(s) over which costs and consequences are being evaluated	70	0
	Discount rate (2)	If used, report the choice of discount rate(s) used for costs and outcome^c^	63	75
		Describe why these are appropriate^c^	25	75
	Outcomes (2)	Describe what outcome was used as the measure of benefit in the evaluation^a^	86	43
		Discuss why it was most appropriate	56	43
	Estimating resources and costs (3)	Describe primary data collection methods and rationale	44	21
		Describe how and why secondary data sources have been chosen and located	37	0
		Describe how opportunity costs have been accounted for	25	0
	Currency, price date, and conversion (2)	Report the dates of the estimated resource quantities and unit costs	41	0
		Describe methods for adjusting estimated unit costs to the year of reported costs if necessary^c^	25	68
		Describe methods for converting costs into a common currency base and the exchange rate	44	17
	Choice of model (2)	Describe and give reasons for the specific type of decision‐analytical model used	52	11
		Provide a figure to show model structure	32	5
	Assumptions (1)	Describe all assumptions underpinning the decision‐analytical model	41	0
	Analytical methods (4)	Describe methods for dealing with skewed, missing, or censored data^b^	13	2
		Describe extrapolation methods and methods for pooling data^b^	15	2
		Describe approaches to validate or make adjustments to a model^b^	11	2
		Describe methods for handling population heterogeneity and uncertainty	22	0
Results	Study parameter (4)	Report the values, ranges, references, and, if used, probability distributions for all parameters	32	0
		Report reasons or sources for distributions used to represent uncertainty where appropriate	21	3
		Report the results of sensitivity analysis^b^	19	2
		Providing a table to show the input values is strongly recommended	43	0
	Incremental costs and outcome (2)	For each intervention, report mean values for the main categories of estimated costs and outcomes of interest, as well as mean differences between the comparator groups	58	51
		If applicable, report incremental cost‐effectiveness or other relevant ratios^c^	42	81
	Characterizing uncertainty (1)	Describe the effects of uncertainty for all input parameters and the estimated incremental cost and incremental effectiveness parameters, together with the impact of methodological assumptions (such as discount rate, study perspective)^b^	10	6
	Characterizing heterogeneity (1)	If applicable, report differences in costs, outcomes, or cost‐effectiveness that can be explained by variations between subgroups of animals with different baseline characteristics or other observed variability in effects that are not reducible by more information	40	8
Discussion	Study findings (1)	Summarize key study findings and describe how they support the conclusions reached^a^	90	0
	Limitations and generalisability (1)	Discuss limitations and the generalisability of the findings	35	0
	Current knowledge (1)	Discuss how the findings fit with current knowledge	50	0
Other	Source of funding (1)	Describe how the study was funded and the role of the funder in the identification, design, conduct and reporting of the analysis	42	0
	Ethical approval (1)	Describe ethical approval process where primary data collection is used^b^	10	21

^a^ Indicates parameter was included in at least 80% of papers for which that parameter was relevant.

^b^ Indicates parameter was included in at most 20% of papers for which that parameter was relevant.

^c^ Indicates parameter not relevant for at least 60% of articles.

Validation of the scoring was performed by GL, who independently scored 10% (randomly selected) of the papers blind to PC's scores; and BH, who independently scored a further three papers. Overall scores for each paper were compared with *R* ^2^ values using Microsoft Excel. Agreement between scorers was calculated as a percentage for each item in the quality assessment in R (R Core Team, 2019).

## 3 RESULTS

### 3.1 Literature search

**FIGURE 1:**
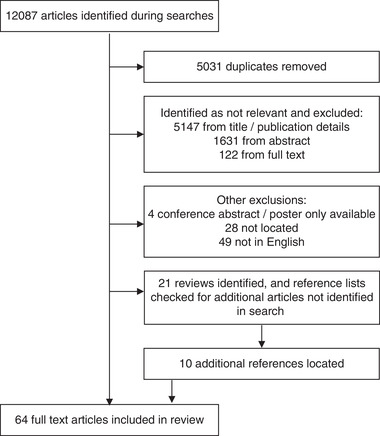
Flow diagram showing the reasons for exclusion of articles from the study. The references included in the review are included as a separate list (see List S2).

Sixty‐four articles were included in the final analysis, from 12,087 that were identified in the searches described in Section 2. The process through which the final articles were selected for inclusion is described in Figure 1.

### 3.2 Data extraction

**FIGURE 2:**
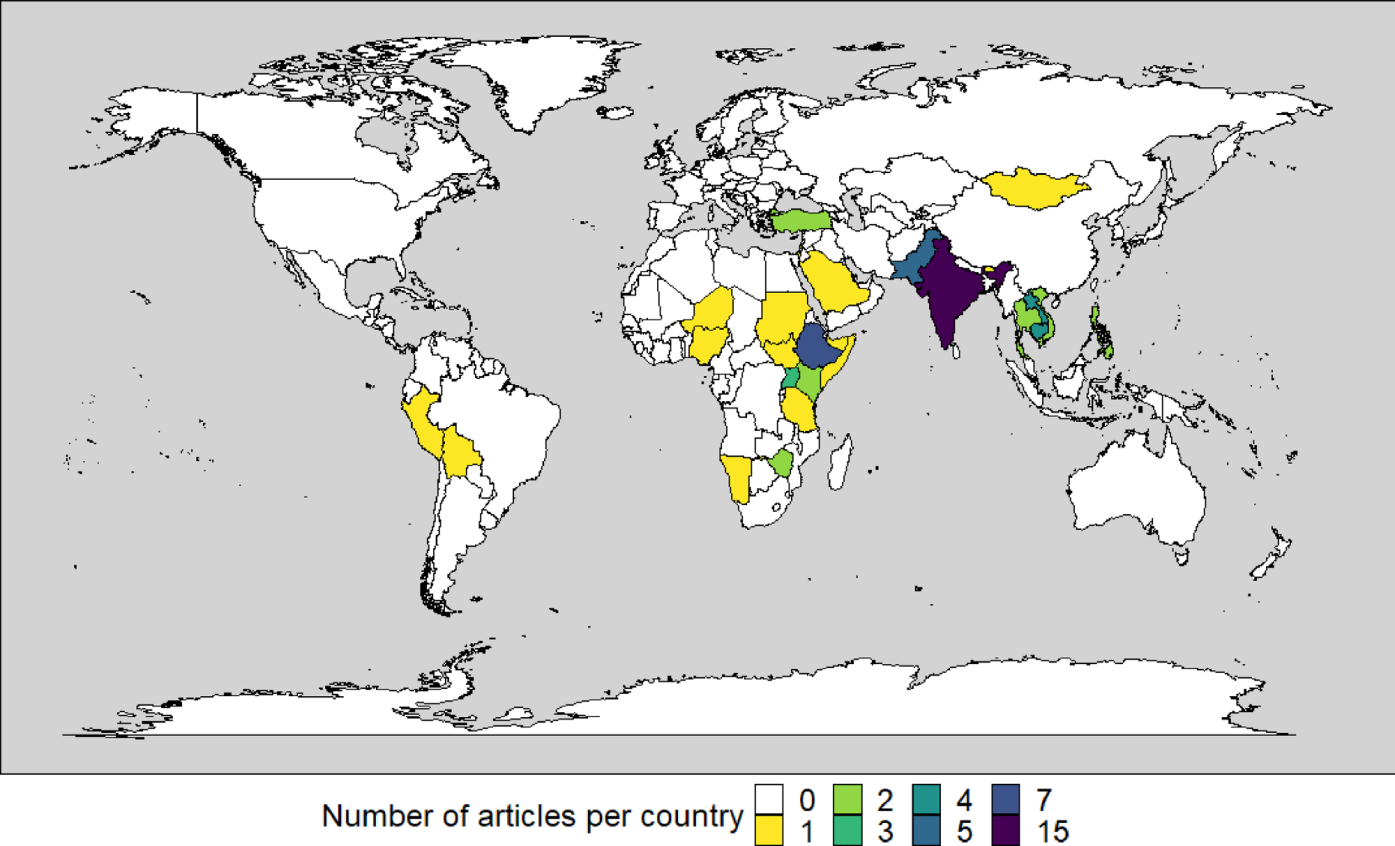
Geographic distribution of articles included in analysis (one paper depicted analysis for the South American region, without identifying specific countries and is not included on this map).

An extended table including data extracted from each paper is included in supporting information (Table S1). The articles included described results for 26 countries (Figure 2). All but one described results from one country; Astudillo and Auge de Mello (1980) depicted analysis across the South American region.

#### 3.2.1 Economic methods

Most articles described deterministic economic impact of disease (58%), often accounting for a selection of costs associated with direct losses at farm or household level (Table 2). None of the articles calculating impact of disease used stochastic methods. Where disease control options were evaluated, 15 out of 25 articles included some elements of stochasticity. Econometric methods were used in two articles; these included the use of regression models (Casey‐Bryars et al., 2018) and error correction models (Abao et al., 2014).

**TABLE 2:** Description of methods used for economic analysis of endemic foot‐and‐mouth disease (FMD) in the articles included in the systematic review. Impacts are classified as direct or indirect according to Knight‐Jones and Rushton (2013)*. ‘Full sensitivity analysis’ means that all input variables were included in sensitivity analysis. ‘Selected sensitivity analysis’ means that selected input variables were included in sensitivity analysis.

Description of methods	Number of articles
Economic impact of disease	37
Deterministic summation of selected costs associated with direct impact of disease	25
Deterministic summation of selected costs associated with indirect impact of disease	2
Deterministic summation of selected costs associated with direct and indirect impact of disease	10
Economic evaluation of disease control	25
Deterministic summation of selected costs associated with direct impact of disease compared*post hoc* to a stated cost of vaccination	5
Ratio of the cost of different disease control programmes to stochastic summation of selected costs associated with direct impact of disease	1
Ratio of vaccination cost to stochastic calculation of selected costs associated with direct impact of disease	1
Deterministic calculation of BCR and/or net benefits using selected direct and indirect impact of disease with selected sensitivity analysis	2
Deterministic partial budget model evaluating vaccination with selected sensitivity analysis	2
Deterministic calculation of BCR, NPV and IRR using selected costs associated with direct and indirect impact of disease	1
Stochastic model evaluating cost effectiveness of different protocols for export using selected indirect impact of disease with full sensitivity analysis	1
Stochastic partial budget model evaluating vaccination with selected sensitivity analysis calculating BCR and NPV	1
Stochastic model evaluating vaccination programme to calculate BCR with full sensitivity analysis	1
Stochastic multiyear model evaluating different eradication/vaccination programmes to calculate NPV and BCR	4
Stochastic multiyear model evaluating vaccination programme to calculate NPV and BCR with selected sensitivity analysis	1
Stochastic multiyear model evaluating vaccination programme to calculate NPV and BCR with full sensitivity analysis	2
System dynamic cost‐benefit model evaluating export scenarios	1
System dynamic cost‐benefit model evaluating trade scenarios with full sensitivity analysis	1
Macroeconomic modelling using a social accounting matrix with a computable general equilibrium algorithm	1
Econometrics	2
Market evaluation of pork and poultry prices following FMD outbreak using an error correction model and historical decomposition of price innovations	1
Micro‐econometric analysis of selected costs associated with direct and indirect impacts of disease	1

Abbreviations: BCR, benefit cost ratio; IRR, internal rate of return; NPV, net present value. *Direct impacts are thoose caused by “reduced production and changes in herd structure”. Indirect impacts are those caused by “costs of FMD control, poor access to markets and limited use of improved technologies”.

#### 3.2.2 Methodological components

Thirty‐six percent of articles used multiple levels of analysis to calculate results (Table 3). Twenty‐one (33%) studies produced national estimates; 14 (22%) regional estimates. Of those, six (17%) produced their estimates based on scaling‐up household estimates; 23% on scaling up animal‐level estimates. Cattle were the species most often considered (in 80% of articles); 28 articles (44%) considered more than one species (Table 3). Of those that included more than one species, 14 included large ruminants only (cattle, buffalo, yak); 12 included both large ruminants and small ruminants/pigs (cattle, buffalo, camels, goats, sheep, pigs); and one study included only small ruminants (goats, sheep).

**TABLE 3:** Characteristics of studies described in the analysis.

Study characteristic		Number of studies (percentage)
Level of analysis^a^	Animal	20 (31)
	Household^#^	21 (33)
	Local	12 (19)
	Regional	14 (22)
	National	21 (33)
	International	1 (2)
	Multiple levels	23 (36)
Type of data collection	Primary	29 (45)
	Secondary	19 (30)
	Primary and secondary	10 (16)
	Not described	6 (9)
Time perspective^a^	Ex ante	46 (72)
	Ex post	23 (36)
Time horizon	<1 year	12 (19)
	1 year	21 (33)
	>1 and ≤5 years	6 (9)
	>5 years	18 (28)
Species^a^	Cattle	51 (80)
	Buffalo	39 (61)
	Goats	9 (14)
	Sheep	7 (10)
	Pigs	6 (9)
	Yak	1 (2)
	Camels	1 (2)
	More than one species	28 (44)

^a^ indicates that a single article can have multiple characteristics within a category; therefore, the sum of the articles will exceed 64 and the sum of percentages will exceed 100.

^#^ The level of ‘household’ included analyses described as at ‘farm’ or ‘herd’ level.

Most studies considered *ex ante* results (72%), of which five (8%) also included *ex post* analysis. Most studies had a time horizon of one year or less; 38% of studies produced multiyear results (Table 3). A mixture of primary and secondary data collection methods was used, although these were often poorly described. Forty‐four percent of articles that used primary data and 37% of articles that used secondary data described their data collection methods adequately (Table 1). Although several papers reported the economic impact of other diseases, these results were not integrated to produce combined figures.

### 3.3 Quality assessment

**FIGURE 3:**
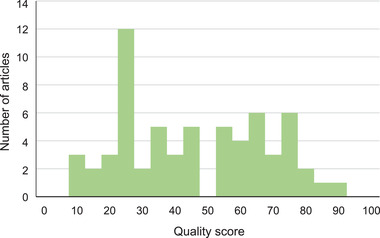
Histogram showing the distribution of quality scores assigned to articles in the systematic review.

The median quality score calculated was 41 (range 8–86; Figure 3). From the 42 parameters assessed, seven parameters were included correctly in at least 80% of papers for which that parameter was relevant: three in the title and introduction, three in the methods and one in the discussion sections (Table 1). Seven parameters were included in at most 20% of papers for which that parameter was relevant: four in the methods and two in the results sections; and one encompassing ethical approval (Table 1). Six parameters were not relevant for at least 60% of papers: five in the methods section and one in the results section (Table 1). Changes in the denominator were not correlated with quality score awarded (*R* ^2^ = 0.17; Figure S3).

#### 3.3.1 Validation of quality assessment

**FIGURE 4:**
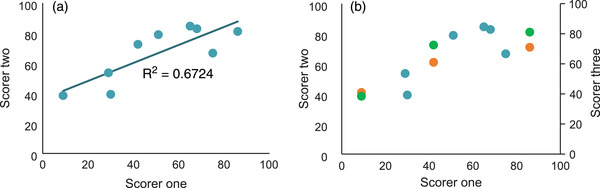
(a) Correlation between total paper scores calculated by two scorers using Pearson's Correlation Coefficient. (b) The same data are displayed as in graph (a), but with the three scores awarded by BH added as orange dots. The green dots identify the same three papers from the original graph.

**FIGURE 5:**
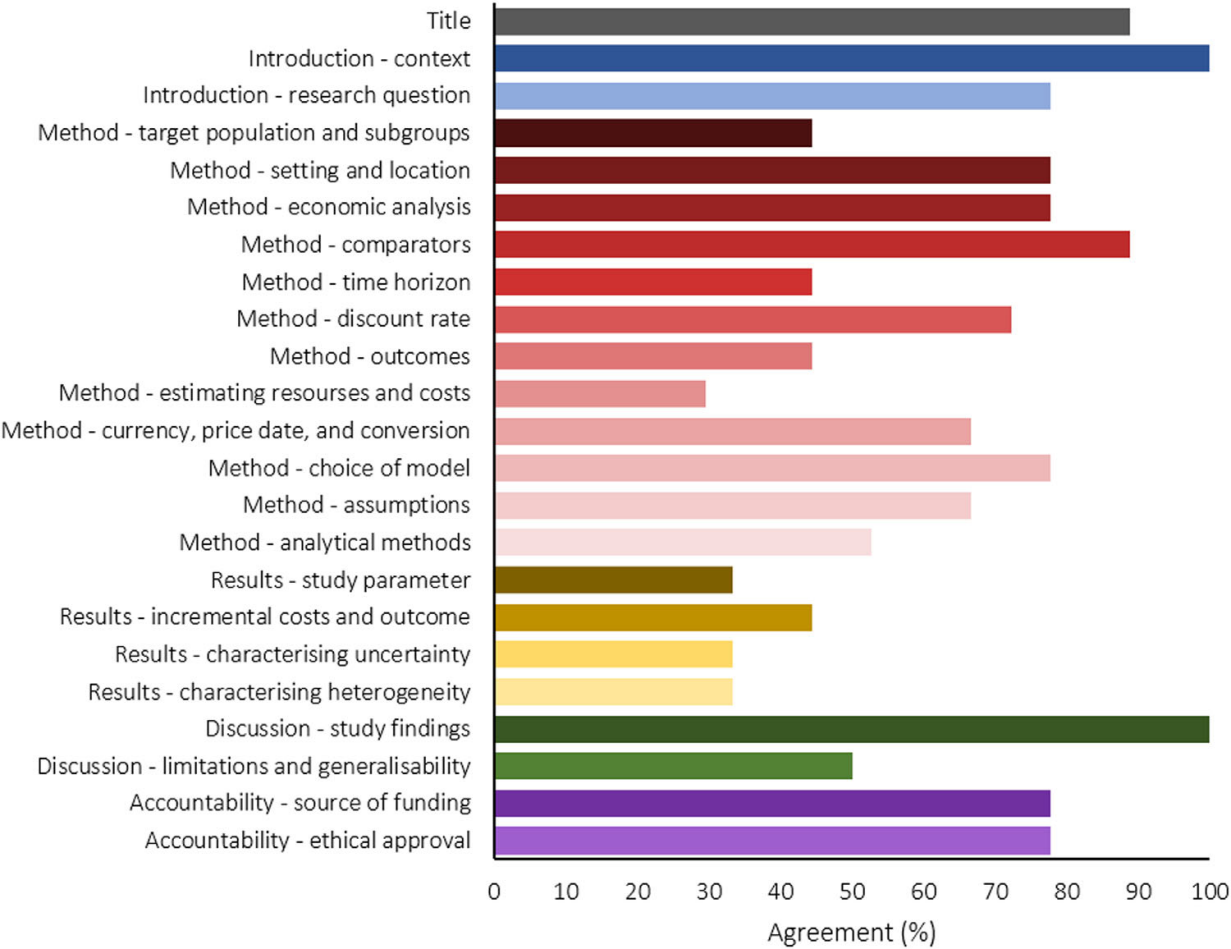
Agreement between scorers by quality assessment parameter for nine papers. *Y*‐axis titles indicate the subgroup under which each parameter is categorized, as described in Table 1. Values shown are mean values for each subgroup. Title represented in grey scale, Introduction represented in shades of blue, Methods represented in shades of red, Results represented in shades of yellow, Discussion represented in shades of green and Accountability in purple shades.

**FIGURE 6:**
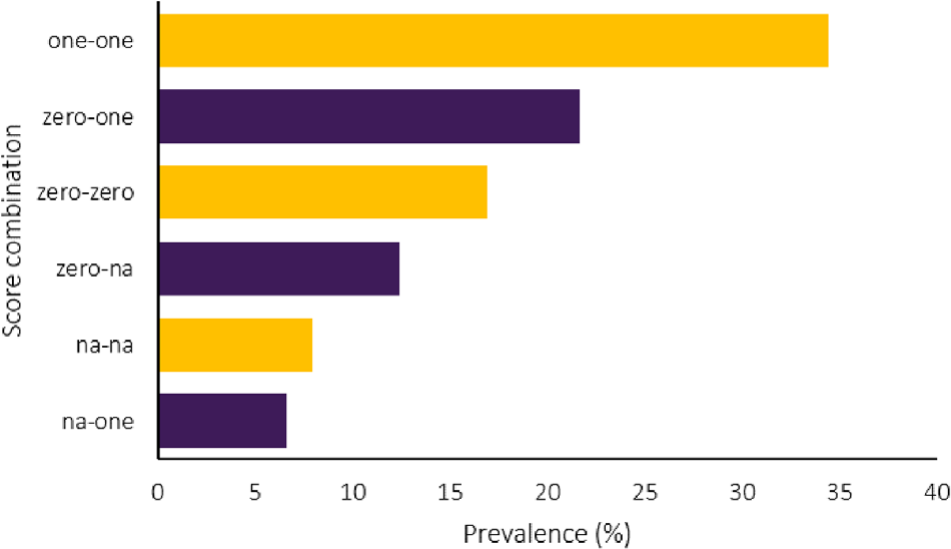
Graph showing frequency of score combinations across all parameters. Score combinations consist of each scorer's result: ‘one’ indicates that the scorer identified that a parameter was included correctly. ‘Zero’ indicates that the scorer identified that a parameter was not included correctly. ‘na’ indicates that the scorer identified that a parameter was not relevant. For example: ‘na‐zero’ indicates that one scorer thought that a parameter was not relevant, and the other scorer thought that parameter was not included correctly. Agreement where both scorers identified a parameter in the same category is represented by yellow bars (e.g., ‘one‐one’, ‘zero‐zero’ and ‘na‐na’).

There was moderate correlation in scoring between PC and GL for nine papers (*R* ^2^ = 0.67, Figure 4a). The results section had the most disagreement between these two authors’ scores, with only one parameter having agreement over 50% (Figure 5). Additionally, some parts of the methods showed low agreement (target populations and subgroups, outcomes, estimating resources and costs and analytical methods). Agreement was seen in other parts of the methods (setting and location, economic analysis, comparators, discount rate and choice of model), as well as the introduction section. On a parameter basis, there was 51.3% agreement between scorers (Figure 6); disagreement occurred most frequently when one scorer thought a parameter had been correctly included and the other scorer did not (22%), followed closely by disagreement because one scorer thought a parameter was not applicable (19%).

The third scorer (BH) evaluated three papers (Figure 4). When the scores from those three papers were combined (from all three scorers), for 38% of parameters agreement between all three was achieved; for 54% of parameters there was agreement between two scorers, and for 8% of parameters there was no agreement. When at least one scorer felt that a parameter was redundant, it was less likely that all scorers would be in agreement (*χ* ^2^ = 23.2, *p *< 0.0001).

## 4 DISCUSSION

This review sets out a comprehensive analysis of the methods used in evaluating the impacts of endemic FMD. The results show that there are discrepancies and diversity in how methods used in these articles are reported, and that this has consequences for the robustness, relevance and use of these results.

### 4.1 Economic methods used in studies assessing endemic FMD

Many methods were not grounded in established practice of animal health economics. Instead, authors often simply summed up a selection of identified economic costs: for example, using results produced at household or animal level and limited direct impacts (e.g., milk yield reduction and mortality) to calculate national impact figures by multiplying with population data, assuming a linear relationship and ignoring heterogenicity among producers.

Even when using accurate input data, the lack of accounting for economic or social influences of scale in vaccination programmes, such as herd immunity or decreased cost of vaccines per unit when purchased in bulk, could result in misleading cost estimates. Few papers included disaggregation of public and private costs, or benefits, of FMD control. Another shortcoming can relate to the time period of analysis where single year or outbreak results are used to produce estimates of disease impact, or disease control programme efficiency. When impacts are measured on an outbreak basis, it is important to define how the outbreak's beginning and end are identified, often not articulated in the articles within this review. This should include the time span for that outbreak, so that production indices, for example, the amount of milk yield forgone, can be placed into context alongside other outbreak scenarios. Moreover, long‐term changes in herd and market dynamics may not be accounted for in these short‐term analyses. An analysis from beef cattle in Bolivia showed that decreased fertility parameters caused by FMD, but not immediately obvious during an outbreak, resulted in losses 4–6 years afterwards (Rushton, 2008).

Additionally, some studies fail to acknowledge implicit assumptions that may cause misleading results and conclusions. Examples include assuming 100% vaccine effectiveness and even access to markets across producers if the disease is controlled, regardless of location and production system. Very few analyses included an assessment of how milk yield losses were influenced by stage of lactation cycle, although this may be less relevant in low‐yielding systems where lactation curves are long and flat (Mrode et al., 2021). Many studies rely on parameters reported by farmers (instead of actual measurements) with associated recall and reporting biases. How bias was considered was not explicitly included as a parameter in the quality appraisal framework. However, only 42% of articles described how the study was funded, 10% described a process of ethical review and 35% included discussion of study limitations. An important consideration in the design of any future quality assessment framework would be how to assess conflicts of interest, or bias associated with a study's objectives.

Stochastic methods were underutilized in the articles included in the review. Stochasticity allows the use of probability distributions to account for variability and uncertainty in input variables over time, resulting in more useful economic estimates. However, stochasticity necessitates more complex calculations, and software which may not be accessible to some authors.

The histogram of the quality assessment scores revealed two main populations (Figure 3). Although not a universal truth, many of the articles with higher scores were produced through collaboration between authors from the study site and (a limited pool) of authors with expertise in veterinary economic analyses and published in higher impact journals, potentially suggesting a more robust peer review process. Figure 2 shows that these studies originate from a limited geographical range, and further examination identifies that some authors are included on many papers. Many countries with endemic FMD, especially in Africa, are not represented at all. The challenges associated with achieving equity within global health research and peer‐review academic publishing are multifaceted and have been well articulated elsewhere (Bhakuni & Abimbola, 2021). These challenges can begin to be addressed through collaboration between national veterinary departments and international agencies supporting progressive FMD control.

It is important that policy makers understand how to commission new research that is targeted to their context and draw appropriate conclusions from pre‐existing research. For countries progressing through the PCP‐FMD, this is most relevant when completing the impact section of their risk‐based strategic plan (FAO et al., 2020). A process for formal support that focuses on understanding how to use economic studies as the risk‐based strategic plan is being written could result in increasing the robustness of disease control programmes.

### 4.2 Reporting in animal health economics

Two main components are important in evaluation of an academic paper. The first lies in the methods used to implement the research and the second in the transparency of how those methods are reported. In this review, the quality of the reporting of methods was assessed and found to contain wide variation. This supports previous conclusions that described difficulties in using published data to evaluate the economic impact of endemic FMD (Knight‐Jones et al., 2017).

Validation of the quality assessment tool was not perfect. There are likely to be several reasons for this. As the CHEERS tool is taken from human health economics, the parameters measured do not all directly correspond to aspects of animal health economics, resulting in a challenge for consistent scoring. It became apparent that the CHEERS tool was intended to evaluate studies that evaluated economic efficiency, whereas several of the studies included in this review focused on economic impact, and a few used econometric methods, both of which were not clearly within the tool's remit. This led to inconsistencies in evaluating whether a parameter was applicable to a study or not, ultimately leading to variations in score. Additionally, a binary scoring system requires additional support to be applied consistently, as when a parameter is incompletely reported within a study, the degree by which it is incompletely reported may contain some subjectivity, leading to scoring discrepancy. The results reported in this review are a starting point to further demonstrate the need for a quality assessment tool for economic studies in animal health, and the challenges faced when developing or adapting an assessment tool.

Overall, the terminology used throughout the articles identified was inconsistent (e.g., cost, loss or impact being used to describe the same thing in different papers). Developing and disseminating standardized definitions and protocols for peer‐reviewed studies of economic impact of animal diseases, for example, along the lines of the STROBE guidelines for epidemiological studies, could support improved estimation of their socioeconomic impacts, as well as supporting both authors and peer reviewers in their academic dissemination. These guidelines could be codesigned and owned by inter‐governmental bodies responsible for disease control: including FAO, OIE, African Union—Interafrican Bureau for Animal Resources (AU‐IBAR), ASEAN Coordinating Centre for Animal Health and Zoonoses (ACCAHZ) and the Pan American Health Organization (PAHO), as well as other relevant stakeholders. This would facilitate the integration of animal economics more widely into policy making. Future work could concentrate on validating these requirements and exploring how such a framework could be applied to FMD‐free countries as well as to other diseases.

### 4.3 Conclusions

In a review of this nature, it is important to acknowledge that no single analysis will provide absolute answers about the economic impact of disease. These impacts will be context‐specific, dependent on both the farming and animal health systems present. Confounding from other diseases and the impact of exogenous environmental or socioeconomic events (e.g., drought or the coronavirus pandemic) will influence these impacts in unpredictable ways. Therefore, it is especially important to report economic methods and associated assumptions fully, because understanding how calculations and values have been arrived at is crucial for their application. Improving the economic literacy of veterinary epidemiologists (and therefore peer reviewers) could result in an improvement in the quality of published studies. Guidelines for quality control as discussed above would support this.

Although an original aim of this work was to enable identification of economic analysis methods that are robust and result in the most applicable measures of disease impact and economic efficiency of control, this was challenging considering our results. Therefore, it is difficult to assess how useful results presented in these studies are when planning and budgeting FMD‐control programmes in endemic contexts. Poor quality economic analyses are unlikely to result in accurate estimation of economic impact, which may have consequences for resource allocation and prioritization in disease control programmes. There are tangible opportunities to increase the quality and therefore value of economic analyses by aligning these academic endeavours with the policy tools used by governments to progress through the PCP‐FMD. Capitalizing on these offers an evidence‐to‐policy pathway, maximizing the impact of future economic studies of endemic and epidemic FMD as well as other diseases affecting production in livestock species.

## CONFLICT OF INTEREST

The authors declare no conflict of interest.

## ETHICS STATEMENT

The authors confirm that the ethical policies of the journal, as noted on the journal's author guidelines page, have been adhered to. No ethical approval was required as this is a review article with no original research data.

## Supporting information

Table S1

List S2

Figure S1

## Data Availability

The data that support the findings of this study are available from the corresponding author upon reasonable request.
