# Supplementary material for: A systematic review of the methods used to analyze the economic impact of endemic foot‐and‐mouth disease
Source: Transbound Emerg Dis. 2022 Jun 20;69(5):e2249–60. doi: 10.1111/tbed.14564 (PMC9795869; doi:10.1111/tbed.14564)
Supplement: Supplementary file 1 — Table S1 [file TBED-69-e2249-s002.docx]

| **Table S1**. Details of articles included in the review | | | | | | |
| --- | --- | --- | --- | --- | --- | --- |
| **Title** | **Country, year** | **Objective of analysis** | **Categorisation of methods** | **Details of methods** | **Data collection** | **Population;**  **Geographical level of analysis;**  **Time perspective;**  **Time horizon** |
| Cost and effectiveness analysis of two foot-and-mouth disease vaccination procedures ^1^ | South America, 1980 | To "determine if the cost of [oil-adjuvanted vaccine] justifies its adoption and utilisation [compared with aluminium hydroxide-saponin]" | *Economic impact of disease*: deterministic summation of selected costs associated with indirect impact of disease | Cost of vaccination: Compared both vaccinations including market cost of vaccines, operating costs for vaccination, number of animals to be vaccinated, vaccine efficacy, age distribution of herd | *Secondary*: previously published literature, national animal health service | Cattle;  International;  Prospective;  1 year |
| Cost of vaccinating against foot and mouth disease in Peru ^2^ | Peru, 1981 | Identify the costs of the vaccination programme in Peru | *Economic impact of disease*: deterministic summation of selected costs associated with indirect impact of disease | Cost of vaccination: including employed personnel, short term consumables, externally sourced services, taxes, depreciation and vaccine | *Secondary*: collected from the national FMD control programme | Cattle;  National;  Retrospective;  3 months |
| An outbreak of Foot and mouth disease, and its socio-economic effects, in a herd of pigs in Nairobi, Kenya ^3^ | Kenya, 1991 | Report economic effects of FMD on a pig farm | *Economic impact of disease*: deterministic summation of selected costs associated with direct impact of disease | Cost incurred due to disease: weight loss, mortality, treatment and disinfection, slaughterhouse closure, employee costs | *Primary*: farmer questionnaire | Pig;  Household;  Retrospective;  Not described |
| Investigation on epidemiology and economic losses of major livestock diseases in District Gujarat ^4^ | Pakistan, 1992 | "Present a clear picture of incidence and economic losses due to livestock diseases" | *Economic impact of disease:* deterministic summation of selected costs associated with direct impact of disease | Cost incurred due to disease: loss of milk yield | *Primary*: farmer questionnaires | Cattle, buffalo, goats, sheep;  Regional;  Retrospective;  1 year |
| Intensive surveillance of foot-and-mouth disease in selected livestock populations in Haryana ^5^ | India, 1993 | not clearly stated: calculate economic losses due to FMD through an intensive surveillance programme | *Economic impact of disease*: deterministic summation of selected costs associated with direct impact of disease | Cost incurred due to disease: summation of milk and draught power losses | *Primary*: farmer questionnaires *Secondary*: unclear | Cattle, Buffalo;  Household, local;  Retrospective;  "Per outbreak" |
| Economic value of some non-milk losses caused by foot-and-mouth disease (FMD) in India ^6^ | India, 1994 | "Estimate some non-milk losses caused by foot and mouth disease of livestock in India" | *Economic impact of disease*: deterministic summation of selected costs associated with direct impact of disease | Cost incurred due to disease: draught power, loss due to permanent disability, mortality, treatment costs | *Primary*: farmer questionnaires  *Secondary*: government records | Cattle, buffalo  Animal, national  Retrospective  1 year |
| Estimated damage to the Israeli dairy herd caused by foot and mouth disease outbreaks and a cost/benefit analysis of the present vaccination policy ^7^ | Israel, 1994 | "Evaluate and compare the costs of the measures [currently in place] to control FMD in Israel in view of the possibility that vaccination ... may cease" | *Economic evaluation of disease control*: ratio of vaccination cost to deterministic summation of selected costs associated with direct impact of disease | Summation of loss from national dairy herd (milk loss, calf mortality, veterinary costs) compared by vaccination status and divided by cost of vaccination | Not described | Dairy cattle;  Animal, national;  Retrospective, prospective;  1 year |
| Economic value of milk loss caused by foot-and-mouth disease (FMD) in India ^8^ | India, 1995 | "[Review] the economic value of the losses in milk production of cows and buffaloes suffering from [FMD]" | *Economic impact of disease:* deterministic summation of selected costs associated with direct and indirect impacts of disease | Cost incurred due to disease: direct reduction in milk loss, losses due to delayed conception and reduction due to abortions. Economic value estimated by potential export value and domestic economic surplus lost | *Primary*: farmer questionnaires *Secondary*: government records | Cattle, buffalo;  Animal, national;  Retrospective;  1 year |
| An Economic Evaluation of the Impact of Foot and Mouth Disease and its Control in Bhutan ^9^ | Bhutan, 1995 | "evaluate the costs and benefits of the control measures" currently in place (universal vaccination) and a new control policy (selective vaccination) | *Economic evaluation of disease control:* deterministic calculation of benefit cost ratio, net present value, internal rate of return using selected costs associated with direct and indirect impact of disease. | Livestock Productivity Efficiency Calculator used to calculate productivity with and without FMD, then building estimate under targeted vaccination strategy to calculate benefit cost ratio, net present value, internal rate of return | *Secondary*: author knowledge, personal communication, government datasets, previously published literature | Cattle, yak;  National;  Retrospective, prospective;  15 years |
| Statistical model for estimating losses due to foot-and-mouth disease in India ^10^ | India, 1997 | "estimat[e] the economic losses caused by morbidity and mortality and gain derived due to introduction of vaccination programme" | *Economic evaluation of disease control:* ratio of vaccination cost to stochastic calculation of selected costs associated with direct impact of disease | Stochastic epidemiological model incorporating costs of milk reduction, meat reduction, cost of medicine, cost of labour, infrastructure cost associated with disease. Vaccine cost not incorporated into model but compared with model output | *Secondary*: not described | Cattle;  Unclear: household level analysis with national conclusions;  Prospective;  6 periods of infection |
| The role of small ruminants in the epizootiology of foot and mouth disease in Saudi Arabia with reference to the economic impact of the disease on sheep and goats ^11^ | Saudi Arabia, 1998 | Not clearly stated: Assess the cost of vaccination against the cost of mortality in lambs and kids due to FMD | *Economic evaluation of disease control:* ratio of vaccination cost to deterministic summation of selected costs associated with direct impact of disease | Cost benefit: summation of cost of kid/lamb mortality annually against cost of vaccination | *Secondary*: not described | Goats, sheep;  National;  Retrospective;  1 year |
| The economic impact of foot and mouth disease and its control in South-East Asia: a preliminary assessment with special reference to Thailand ^12^ | Thailand, 1999 | "Determining the economic impact of FMD and the control of the disease" in Southeast Asia using Thailand as an example | *Economic evaluation of disease control:* stochastic multiyear model evaluating different vaccination programmes to calculate benefit cost ratio, net present value | Epidemiological model integrated with economic model simulating current baseline and 3 different vaccine scenarios (with different frequency of vaccination). Benefit cost ratio, net present value calculated. Costs associated with implementation of control measures, surveillance and disease control programme and swine vaccination included. Benefits include production losses, control cost savings and increased exports | *Secondary:* previously published data | FMD-susceptible species;  National;  Prospective;  10-50 years |
| The economic impact of foot and mouth disease control and eradication in the Philippines ^13^ | Philippines, 2002 | "Assessment of the economic impact of FMD and the proposed control programme and other options" | *Economic evaluation of disease control:* stochastic multiyear model evaluating different eradication programmes to calculate benefit cost ratio, net present value | Stochastic analysis used to calculate net present value and benefit cost ratios for three different eradication scenarios that differ in length. Costs defined as disease surveillance, public awareness, animal movement control, vaccination, containment. Benefits defined as (i) saving from treatment of infected animals, vaccination and outbreak containment (ii) production losses: mortality, weight loss, milk losses, abortions, poor reproductive performance, draught power loss, manure losses (iii) market effects: movement controls, price fluctuations and consumer scares (iv) enhanced export opportunities | *Secondary:* previously published data and national datasets | FMD-susceptible species;  National;  Prospective;  30 years |
| The impact and poverty reduction implications of foot and mouth disease control in southern Africa with special reference to Zimbabwe ^14^ | Zimbabwe, 2003 | "establish cost-effective disease management strategies that reflect national economic development priorities, including poverty reduction" | *Economic evaluation of disease control:* stochastic multiyear model evaluating different vaccination programmes to calculate benefit cost ratio | Incremental costs and benefits for (i) a reduced FMD free zone with current level of FMD control: costs of conversion of quarantine zone to surveillance and a small FMD-free zone, benefits of decreased risk of FMD suspending trade; (ii) improved FMD control: costs of strengthened veterinary services and repair to fencing, benefits of reduced probability of FMD outbreaks and reduced preventative vaccination/outbreak management costs (iii) relaxed FMD control: costs of less investment in vet services and FMD control a with negative benefits of increased outbreaks and production losses, loss of expert revenues and increased private vaccination. Monte Carlo stimulations | *Primary:* farmer surveys *Secondary:* not described | Cattle;  Household, national;  Prospective;  25 years |
| Prevalence of major livestock diseases in north Waziristan agency ^15^ | Pakistan, 2003 | "Estimate economic losses inflected [sic] by major livestock diseases in buffaloes, cattle, goats, and sheep in North Waziristan Agency" | *Economic impact of disease*: deterministic summation of selected costs associated with direct impact of disease | Cost incurred due to disease calculated from market price of animals, treatment cost, loss in animal product | *Primary:* farmer interviews/ questionnaires  *Secondary:* not described | Cattle, buffalo, goats, sheep;  Regional;  Retrospective;  1 year |
| Financial Impact Assessment of Foot and Mouth Disease in Large Scale Farms in Nakuru District, Kenya ^16^ | Kenya, 2005 | "estimate the direct impact of SAT1 FMD outbreaks in four large scale farms in Nakuru district" | *Economic impact of disease:* deterministic summation of selected costs associated with direct impact of disease | Cost incurred due to disease: treatment costs, mortality/culling in cattle and pigs, extra feeding costs, extra labour, extra artificial insemination, milk production losses, extra transport costs | *Primary*: farmer questionnaires | Dairy cattle, pigs;  Local;  Retrospective;  1 outbreak |
| Evaluating equity impacts of animal disease control the case of foot and mouth disease in Zimbabwe ^17^ | Zimbabwe, 2005 | "report extensions to the traditional BCA framework to address ... equity impacts as part of an evaluation of improved FMD control in Zimbabwe recently undertaken by Perry et al." | *Economic evaluation of disease control:* macroeconomic modelling using a social accounting matrix with a computable general equilibrium algorithm. | Costs and benefits from previous study disaggregated by household income and private and public sector, following by macroeconomic modelling using a social accounting matrix with a computable general equilibrium algorithm. Baseline scenario compared to different degrees of export ban from EU. Transmission of price shock from beef export market back to / within cattle markets analysed | *Secondary*: previously published data | Cattle;  National;  Prospective;  25 years |
| Economic losses due to mortality and culling of foot and mouth disease affected livestock ^18^ | India, 2006 | "estimate the economic losses due to mortality and culling of FMD-affected animals" | *Economic impact of disease:* deterministic summation of selected costs associated with direct impact of disease | Cost incurred due to disease: mortality, difference between market value before and after disease, loss due to culling. | Not described | Cattle, buffalo, sheep, goat;  Local;  Retrospective;  3 months |
| Monetary losses due to reproductive failures in FMD affected bovines ^19^ | India, 2007 | "Estimate the monetary losses arising due to abortions and repeat breeding / infertility in FMD affected female bovines" | *Economic impact of disease:* deterministic summation of selected costs associated with direct impact of disease | Cost incurred due to disease: impact of abortions (cost of calves and milk loss) and extended calving intervals (loss of offspring and milk loss) | Not described | Cattle, buffalo;  Regional;  Retrospective;  3 months |
| Foot-and-mouth disease vaccination in South Sudan: Benefit-cost analysis and livelihoods impact ^20^ | South Sudan, 2008 | "[present] a BCA of FMD vaccination in an agropastoral area of South Sudan" | *Economic evaluation of disease control:* deterministic calculation of benefit cost ratio using selected costs associated with direct and indirect impact of disease. Selected sensitivity analysis | Mortality costs: acute disease, chronic disease, calves from pregnant cows that die from acute or chronic disease. Milk loss costs: short term reduction due to clinical disease, long term losses due to acute deaths and chronic disease. Costs of vaccination include importation, equipment / cold chain, training, supervision, overheads and administration. Sensitivity analysis for vaccination costs and FMD prevalence | *Primary*: participatory epidemiology  *Secondary*: national datasets | Cattle;  Regional;  Prospective;  1 year |
| Economic impact of FMD in Chazhoor Panchayath ^21^ | India, 2008 | "assess the potential economic impact of an outbreak of FMD" | *Economic impact of disease:* deterministic summation of selected costs associated with direct and indirect impact of disease | Cost incurred due to disease: reduction in milk yield, mortality, abortion, treatment costs, additional labour, loss of draught power, vaccination | *Primary*: farmer questionnaires | Buffalo, cattle;  Local;  Retrospective;  1 outbreak |
| Modelling of economic losses due to some important diseases in goats in India ^22^ | India, 2008 | "[assess] losses due to major diseases in goats in India." | *Economic impact of disease:* deterministic summation of selected costs associated with direct impact of disease | Cost incurred due to disease: losses from mortality, milk losses, cost of loss of live body weight due to increased inter-kidding period, cost of loss of live body weight due to increased occurrence of abortions and opportunity | *Secondary*: government data | Goats;  National;  Retrospective;  15 years |
| Production losses due to endemic foot-and-mouth disease in cattle in Turkey ^23^ | Turkey, 2008 | "[estimate] the endemic foot-and-mouth disease (FMD)-induced production losses in individual dairy and fattening cattle" | *Economic impact of disease:* deterministic summation of selected costs associated with direct impact of disease | Cost incurred due to disease: decreases in milk yield, fertility, premature culling, liveweight loss, loss in expected profit (opportunity costs) | *Secondary*: results from published Delphi expert opinion survey, government data, market data | Cattle;  Animal, national;  Retrospective;  1 outbreak |
| Economic aspects of foot and mouth disease in Bolivia ^24^ | Bolivia, 2008 | "present the … impact of FMD and its control in the extensive beef systems in the tropical lowlands of [Bolivia]" | *Economic impact of disease:* deterministic summation of selected costs associated with direct and indirect impact of disease | Cost incurred due to disease and cost of vaccination: dynamic deterministic model, including herd dynamics, value of affected and healthy animals, fertility impact, morbidity rate, cost of vaccination | *Secondary*: not described | Beef cattle;  Household;  Prospective;  10 years |
| Economic evaluation of important cattle diseases in India ^25^ | India, 2008 | "estimate economic losses due to four important livestock diseases (FMD, HS, BQ, Anthrax) | *Economic impact of disease:* deterministic summation of selected costs associated with direct impact of disease | Cost incurred due to disease: summation of losses from mortality, milk loss and opportunity cost (increased feed requirement, medicine, increased rearing time, permanent disability) | *Secondary*: government records | Cattle;  National;  Retrospective;  15 years |
| A model-based assessment of economic losses due to some important diseases in sheep in India ^26^ | India, 2009 | "Assess economic losses due to important diseases in sheep" (1991 - 2005) | *Economic impact of disease:* deterministic summation of selected costs associated with direct impact of disease | Cost incurred due to disease: Calculated from mortality, wool losses, body weight losses, opportunity cost | *Secondary*: government records | Sheep;  National;  Retrospective;  15 years |
| Commodity-based trade and market access for developing country livestock products: the case of beef exports from Ethiopia ^27^ | Ethiopia, 2009 | "Examine the feasibility of a proposed two-phase SPS certification system designed to enhance beef exports from Ethiopia" | *Economic evaluation of disease control*: system dynamic cost-benefit model evaluating export scenarios | Dynamic cost-benefit model using system dynamics evaluating two different feed rations: enterprise budgets and gross margins, export flows, specific production costs, as well as training costs, capital investments, laboratory diagnostic and vaccine cists; rejection costs, certification costs | *Primary*: key informant interview, feedlot and abattoir surveys *Secondary*: expert opinion | Beef cattle;  Animal;  Prospective;  5 years |
| Limiting the impacts of foot and mouth disease in large ruminants in northern Lao People's Democratic Republic by vaccination: a case study ^28^ | Laos, 2010 | Estimate financial impact of FMD in vaccinated and unvaccinated villages | *Economic evaluation of disease control:* ratio of vaccination cost to deterministic summation of selected costs associated with direct impact of disease | Cost incurred due to disease from mortality, morbidity (treatment costs, delayed weight gain, reduced weight at sale) compared with cost of vaccination | *Primary*: semi-structured questionnaires | Cattle, buffalo;  Animal, local;  Retrospective;  2-3 months |
| Simulative modeling to control the Foot and Mouth Disease epidemic ^29^ | Turkey, 2010 | Simulate infection networks, assess FMD mitigation strategies and study direct costs associated with these | *Economic evaluation of disease control:* ratio of different FMD control programme cost to stochastic summation of selected costs associated with direct impact of disease | Cost effectiveness calculated by dividing the reduction in infected livestock (as modelled by epidemiological model) by cost incurred for each of 6 mitigation that include culling, vaccination and movement bans to different extents / orders | *Secondary*: previously published data and national dataset | Cattle, sheep;  National;  Prospective;  1 year |
| Economic impact of an outbreak of foot and mouth disease in Khartoum State, Sudan ^30^ | Sudan, 2012 | Describe the economic impact of an FMD outbreak in dairy farms in Khartoum State | *Economic impact of disease:* deterministic summation of selected costs associated with direct impact of disease | Cost incurred due to disease: reduced milk yield, treatment costs, mortality | *Primary*: farmer and animal healthcare practitioner questionnaires | Dairy cattle;  Regional;  Retrospective;  1 outbreak |
| Animal disease and livestock-keeper livelihoods in Southern Cambodia ^31^ | Cambodia, 2012 | "The direct costs imposed by FMD on smallholders constitute what proportion of household income? Is disease burden regressive i.e. are costs as a proportion of household income higher for lower income households? How significant are the costs imposed by FMD on affected households?" | *Economic impact of disease:* deterministic summation of selected costs associated with direct impact of disease | Cost incurred due to disease: Direct short terms costs incurred following an outbreak, as a proportion of household income | *Primary*: qualitative interviews and focus groups, participatory epidemiology, quantitative questionnaires | Pigs, cattle;  Household;  Retrospective;  1 year |
| Costs and benefits of foot and mouth disease vaccination in commercial dairy farms in Central Ethiopia ^32^ | Ethiopia, 2012 | "estimate the economic losses of an outbreak and to predict the costs and benefits of vaccination under three scenarios: no vaccination, reactive vaccination and preventive vaccination and two sub-scenarios: treatment and no treatment during an outbreak" | *Economic evaluation of disease control:* stochastic multiyear model evaluating different vaccination programmes. Full sensitivity analysis. | Calculation of Cost incurred due to disease: milk yield reduction, mortality, abortion loss, premature culling, treatment, extra feed, farm and labour, vet visit. Reactive and preventative vaccination scenarios compared | *Primary*: farmer questionnaires and expert opinion  *Secondary*: previously published data | Dairy cattle;  Household;  Prospective;  5 years |
| Assessment of Financial Impact of Foot and Mouth Disease on Smallholder Cattle Farmers in Southern Cambodia ^33^ | Cambodia, 2013 | "estimate the financial impact of an outbreak of FMD on smallholder farmers in southern Cambodia and determine, using a partial budget assessment, the net benefit/ cost of biannual FMD vaccine use by smallholder cattle farmers" | *Economic evaluation of disease control:* deterministic partial budget model evaluating vaccination with selected sensitivity analysis | Financial impact assessment: change in value of cattle due to FMD outbreak, treatment costs, draught animal replacement. Partial budget analysis for biannual vaccination: Additional returns: weight gain in healthy animals; Reduced costs: treatment cost, cost of weight loss, selling dead animals and replacement draught costs; Returns forgone: zero; Extra costs: purchase and administration of vaccine. Sensitivity analysis for cattle value, disease duration, draught replacement, treatment cost, weight loss and FMD incidence. | *Primary*: Farmer questionnaire | Cattle;  Animal;  Prospective;  1 year |
| Estimation of economic losses due to foot and mouth disease in India^34^ | India, 2013 | "evaluation of economic losses considering all possible direct and indirect losses [due to FMD] in five species, viz. cattle, buffalo, goat, sheep and pig" | *Economic impact of disease:* deterministic summation of selected costs associated with direct impact of disease | Cost incurred due to disease: mortality; milk yield reduction; reproductive failure in affected female animals; loss of draught power; growth reduction of new born animals; treatment costs; opportunity costs. | *Secondary*: previously published data and national records | Cattle, buffalo, sheep, goats, pigs;  Animal, national;  Retrospective;  1 year |
| Quantity and value of milk losses due to technical constraints - a case of crossbred cows in north-eastern states of India.^35^ | India, 2013 | "(1) To estimate the quantity and value of milk losses due to technical constraints in crossbred milk production system and (2) prioritization of constraints to ensure higher returns to the public investments in research, development and extension services. " | *Economic impact of disease:* deterministic summation of selected costs associated with direct impact of disease | Cost of milk losses caused by clinical disease (not including fertility impacts/abortions) | *Primary*: farmer questionnaires  *Secondary*: government data | Dairy cattle;  Animal, regional;  Retrospective;  1 year |
| Economic impact of foot and mouth disease outbreaks on smallholder farmers in Ethiopia ^36^ | Ethiopia, 2014 | "direct economic impact of FMD outbreaks on household level in the small- holder livestock farming systems in Ethiopia" | *Economic impact of disease*: deterministic summation of selected costs associated with direct impact of disease | Cost incurred due to disease: reduction in milk yield caused by disease or calf loss, draught power loss, mortality losses | *Primary*: Farmer interviews | Cattle;  Animal, household;  Retrospective;  1 outbreak |
| Seroprevalence of foot and mouth disease (FMD) and associated economic impact on Central Ethiopian cattle feedlots. ^37^ | Ethiopia, 2014 | "determine... economic impact [of FMD] on Central Ethiopian cattle feedlots" | *Economic impact of disease:* deterministic summation of selected costs associated with direct impact of disease | Cost incurred due to disease: calculated from prevalence and average price | Not described | Beef cattle;  Regional;  Retrospective;  1 year |
| Risk assessment and cost-effectiveness of animal health certification methods for livestock export in Somalia ^38^ | Somalia, 2014 | "cost- effectiveness analysis on different health certification protocols for Somali livestock exports for six transboundary diseases" | *Economic evaluation of disease control:* stochastic model evaluating selected indirect costs of disease. Full sensitivity analysis. | Quantitative risk pathway articulated. Cost effectiveness calculated as cost of health certification divided by number of infected animals exported. Monte Carlo simulation. Full sensitivity analysis on input variables. | *Primary*: expert opinion workshop | Cattle, sheep, goats;  Animal;  Prospective;  Not described |
| Prevalence of the major infectious animal diseases affecting livestock trade industry in Ethiopia ^39^ | Ethiopia, 2014 | "estimate the financial losses [from major infectious animal diseases] at Adama-Modjo Quarantine Stations" | *Economic impact of disease:* deterministic summation of selected costs associated with direct impact of disease | Cost incurred due to disease: prevalence, weight and price per kg | Not described | Bulls;  Local;  Retrospective;  6 months |
| Prevalence and economic importance of Foot and Mouth Disease, and Contagious Bovine Pleuropneumonia Outbreaks in cattle in Isingiro and Nakasongola Districts of Uganda ^40^ | Uganda, 2014 | "[determine] the financial losses associated with FMD and CBPP outbreaks in selected case study cattle herds in Isingiro and Nakasongola districts" | *Economic impact of disease:* deterministic summation of selected costs associated with direct and indirect impact of disease | Cost incurred due to disease: vaccination and treatment costs, mortality and weight loss, abortion, reduction in milk yield, reduced manure production, traction power loss, reduced crop production, salvage sales | Not described | Cattle;  Animal, household;  Retrospective;  1 year |
| Financial impact of Foot and Mouth Disease and Contagious Bovine Pleuropneumonia along the cattle marketing chain in selected districts in Uganda.^41^ | Uganda, 2014 | "determine the financial impact of FMD and CBPP along the cattle marketing chain in inland districts of Nakasongola and Nakaseke and International border districts of Isingiro and Rakai in the cattle corridor, Uganda." | *Economic impact of disease:* deterministic summation of selected costs associated with direct and indirect impact of disease | Market losses for farmers: salvage sale losses, milk quarantine losses. Market revenue losses: comparison of income during out breaks and without outbreaks, accounting for normal seasonal variations. | *Primary*: farmer questionnaires, market observation | Cattle;  Regional;  Retrospective;  5 years |
| Foot-and-mouth disease and its effect on milk yield: an economic analysis on livestock holders in Pakistan^42^ | Pakistan, 2014 | Identify economic cost of milk loss caused by FMD and compare to cost of vaccination | *Economic evaluation of disease control:* ratio of vaccination cost to deterministic summation of selected costs associated with direct impact of disease | Milk losses calculated from questionnaire data and then converted to financial figure for all farms, then compared with cost of vaccine and its administration to produce simple benefit cost ratio | *Primary*: farmer questionnaires | Cattle, buffalo;  Local;  Retrospective;  1 year |
| Impact of foot‐and‐mouth disease on pork and chicken prices in Central Luzon, Philippines^43^ | Philippines, 2014 | “Determine the impact of FMD outbreaks on: (1) Central Luzon pork and chicken prices from 1995 to 1998, (2) pork and chicken traders’ margins from 1995 to 1998, and (3) the interrelationship among pork and chicken price series from 1995 to 1996” | Market evaluation of pork and poultry prices following FMD outbreak | Error correction model with historical price decomposition to analyse how pork and chicken process changed following FMD outbreaks compared to their predicted changes | *Secondary*: industry datasets | Pigs / pork;  Regional;  Retrospective;  9 years |
| Assessment of socio-economic impacts of Foot-and-Mouth Disease on Cattle in Twelve Villages of Cambodia^44^ | Cambodia, 2014 | "Estimate the direct and indirect socio-economic costs associated with the outbreaks of FMD as well as of the measures taken by farmers to deal with such outbreaks" | *Economic impact of disease:* deterministic summation of selected costs associated with direct impact of disease | Cost incurred due to disease: summation of time spent collecting feed, treatment, loss of business (draught power for markets), cost of replacement cattle work (draught power for agriculture) | *Primary*: key informant interviews, focus group discussions, household survey | Cattle;  Local;  Retrospective;  1 year |
| Would a Commodity-based Trade Approach Improve Market Access for Africa? A Case Study of the Potential of Beef Exports from Communal Areas of Namibia^45^ | Namibia, 2015 | "[assess] the economic potential of commodity based trade | *Economic evaluation of disease control: s*ystem dynamic cost-benefit model evaluating trade scenarios. Full sensitivity analysis | Systems modelling evaluating six trade scenarios for abattoirs, farmers and feedlots. Includes personnel costs, non FMD veterinary costs, transportation, feed costs, FMD vaccination, steer procurement, feedlot infrastructure, EU approval, meat processing. Sensitivity analysis for different prevalence of FMD, price of cuts, presence of EU approved abattoir and removal of quarantine | *Primary*: key informant interviews *Secondary*: national data sets | Beef cattle;  Regional;  Prospective;  20 years |
| Economic analysis of dairy animal diseases in Punjab: a case study of Faisalabad district ^46^ | Pakistan, 2015 | "[examine] four different diseases with an aim to estimate the economic losses caused by these diseases and potential returns if these diseases are controlled for" | *Economic evaluation of disease control:* ratio of vaccination cost to deterministic summation of selected costs associated with direct impact of disease | Cost incurred due to disease: initial calculation of farm gross margin. Disease costs calculated from reduction in milk yield, treatment costs, losses dues to abortion, weight loss, mortality. Benefit cost ratio calculated from this using expert opinion for cost of control (no information given) | *Primary*: farmer questionnaire*,* expert opinion | Cattle, buffalo;  Animal, household;  Retrospective, prospective;  1 year |
| Financial Impact of Foot and Mouth Disease on Large Ruminant Smallholder Farmers in the Greater Mekong Subregion^47^ | Laos, 2015 | "determine the financial impact of FMD outbreaks between 2010 and 2012 on large ruminant smallholder farmers" | *Economic evaluation of disease control:* deterministic partial budget model evaluating vaccination with selected sensitivity analysis | Cost incurred due to disease: direct losses (mortality, production losses due to morbidity), indirect losses (treatment costs). Disease control: cost of preventative vaccination programme. Sensitivity analysis exploring proportion of household ruminants affected. Partial budget model for biannual vaccination | *Primary*: farmer questionnaires *Secondary*: village vaccination programme records | Cattle, buffalo;  Household;  Retrospective, prospective;  Not described |
| Incidence of important goat diseases and economic losses under field condition ^48^ | India, 2015 | "assess ... economic losses due to some of the deadly disease of goat" | *Economic impact of disease:* deterministic summation of selected costs associated with direct impact of disease | Cost incurred due to disease: mortality, weight loss and treatment costs | *Primary*: farmer questionnaires | Goats;  Animal, regional;  Retrospective;  Not described |
| Economic effects of foot and mouth disease outbreaks along the cattle marketing chain in Uganda ^49^ | Uganda, 2016 | "[assess] the economic costs incurred by the various actors along the cattle marketing chain during FMD outbreaks" | *Economic impact of disease:* deterministic summation of selected costs associated with direct and indirect impact of disease | Cost incurred due to disease: control costs (vaccination and treatment of *Secondary* infections); weight loss; abortion; milk yield reduction, reduced manure production, salvage sales. Compares between period of outbreak and non-outbreak income for various processor actors | *Primary*: focus group discussions, semi-structured interviews, case studies, farmer/trader questionnaires  *Secondary*: government records | Cattle;  Household, regional;  Retrospective;  Not described |
| Cost-benefit analysis of foot and mouth disease control in Ethiopia^50^ | Ethiopia, 2016 | Estimate annual costs of FMD on cattle production systems and ex ante cost benefit analysis of control strategies | *Economic evaluation of disease control:* stochastic multiyear model evaluating vaccination programme to calculate net present value and benefit cost ratio. Full sensitivity analysis | Costs: production (milk, draft, mortality) losses; export losses; control (vaccination, monitoring, outbreak investigation and treatment) costs: baseline (no vaccination programme) compared with ring vaccination, targeted vaccination, preventative mass vaccination. Incremental benefits: avoided production losses, treatment costs, export losses. incremental costs: increased costs - surveillance, outbreak investigation, movement restrictions, vaccination, post vaccination monitoring, staff capacity building and revenue forgone: vaccination relation milk yield loss. Stochastic analysis. Full sensitivity analysis and break-even analysis. Net present value and benefit cost ratio over 10 years. | *Secondary*: previously published data, national datasets | Cattle;  National;  Prospective;  10 years |
| Benefit-Cost Analysis of Foot and Mouth Disease Control in Large Ruminants in Cambodia ^51^ | Cambodia, 2016 | "estimation of the impact of the 2010 FMD epizootic on smallholder farmers; and a benefit-cost analysis (BCA) for improved FMD control through a national biannual vaccination programme to be conducted" | *Economic evaluation of disease control:* stochastic multiyear model evaluating vaccination using selected costs associated with direct and indirect impact of disease to calculate benefit cost ratio | Financial impact per animal weighted based on outcome (survival and replacement draught), morbidity and use. Mote Carlo stimulation used to derive mean values. Large ruminant value chain articulated. National financial impact calculated from incidence and per animal cost. Control costs calculated from vaccine programmes: vaccine, vehicle, equipment, accommodation, personnel costs. benefit cost based on biannual vaccination | *Primary*: trader questionnaires *Secondary*: previously published data, data from development programme | Cattle, buffalo;  Animal, national;  Retrospective, prospective;  5 years |
| Financial impacts of foot‐and‐mouth disease at village and national levels in Lao PDR ^52^ | Laos, 2016 | "determine estimates of these financial impacts as well as analyse the potential benefits to costs of the FMD vaccination programme" | *Economic evaluation of disease control:* stochastic multiyear model evaluating vaccination programme to calculate benefit cost ratio, net present value. Selected sensitivity analysis | Village: household losses (mortality, morbidity and treatment costs). National: total village level losses and cost of outbreak control (vaccines, vaccination delivery, administrative costs, public veterinary budget). Monte Carlo simulation to account for over or underreporting of FMD incidence: Benefits equated to total cost incurred due to disease and preventative vaccination. Benefit cost ratio, net present value calculated sensitivity analysis for incidence of FMD. | *Secondary*: previously published data, national records | FMD-susceptible livestock;  Local, national;  Retrospective;  5 years |
| Household financial status and gender perspectives in determining the financial impact of foot and mouth disease in Lao PDR.^53^ | Laos, 2016 | "to progress the recently published estimates of the financial impact of FMD in the northern uplands of Laos" Determine losses due to FMD infection in different socioeconomic groups | *Economic impact of disease:* deterministic summation of selected costs associated with direct impact of disease | Cost incurred due to disease: mortality, production losses (based on sale values, excluding draught animals), treatment costs (excluding labour) | *Primary*: participatory tools, farmer questionnaires | Cattle, buffalo;  Household;  Retrospective;  Not described |
| Epidemiological analysis and economic impact assessment of foot-and-mouth disease at Landhi Dairy Colony Karachi^54^ | Pakistan, 2017 | "study ... the economic impact of FMD at LDC, Karachi" | *Economic impact of disease:* deterministic summation of selected costs associated with direct impact of disease | Losses calculated from mortality, reduction in milk yield, treatment costs, reduction in sale price/ cull rate. (described as partial budget analysis but not presented in this way) | *Primary*: data collection from farmers | Dairy Buffalo;  Animal, household, local;  Retrospective;  45 days (1 outbreak) |
| Farm Community Impacts of Foot-and-Mouth Disease Outbreaks in Cattle and Buffaloes in Karnataka State, India^55^ | India, 2017 | "farm community impacts of FMD in cattle and buffaloes" Determine short- and long-term losses caused by FMD infection on dairy farms. | *Economic impact of disease:* deterministic summation of selected costs associated with direct impact of disease | Cost incurred due to disease: direct losses: due to decreased milk yield, draught power, treatment costs, extra labour, distress sales, mortality | *Primary*: farmer questionnaires | Cattle, buffalo;  Animal;  Retrospective;  Not described |
| Financial Impacts of Priority Swine Diseases to Pig Farmers in Red River and Mekong River Delta, Vietnam.^56^ | Vietnam, 2017 | "estimate the costs of swine diseases at the farm level" | *Economic impact of disease:* deterministic summation of selected costs associated with direct and indirect impact of disease | Gross margin estimate developed then calculated disease loss as direct losses: mortality, morbidity (abortion, delayed finishing); indirect costs: control costs (treatment, biosecurity, emergency vaccination) and revenue forgone (lower price of emergency selling) | *Primary*: focus group discussions, farmer questionnaires | Pigs;  Household;  Retrospective;  1 year |
| Waves of endemic foot-and-mouth disease in eastern Africa suggest feasibility of proactive vaccination approaches^57^ | Tanzania, 2018 | "quantify the impacts of FMD on livestock production and household decisions" | *Microeconometric analysis:* of selected costs associated with direct and indirect impact of disease | Microeconometric analysis including impact on milk production, number of lactating cows, traction, livestock sales educational expenditures and human health | *Primary*: household questionnaires | FMD-susceptible livestock;  Household;  Retrospective;  Not described |
| Spatio-temporal patterns of foot-and-mouth disease transmission in cattle between 2007 and 2015 and quantitative assessment of the economic impact of the disease in Niger ^58^ | Niger, 2018 | "obtain insights into the... economic costs of [FMD]...in Niger" | *Economic evaluation of disease control:* stochastic model evaluating vaccination programme to calculate benefit cost ratio. Full sensitivity analysis | Economic impacts: acute milk yield reduction and young animal mortality. Cost of control: cost of vaccination and cost of delivery. Monte Carlo stimulation for benefit cost ratio and full sensitivity analysis | *Secondary*: previously published data, national datasets | Cattle;  Animal, household;  Prospective;  Not described |
| Analysis of foot and mouth disease in dairy animals: An assessment of cost and loss from sample farmers ^59^ | India, 2018 | "assess the impacts of foot and mouth disease on farm production and farmers [sic] economy" | *Economic impact of disease:* deterministic summation of selected costs associated with direct and indirect impact of disease | Cost incurred due to disease: treatment, prevention, extra labour; reduction in yield, decreased market value, hide price, working capacity, losses due to abortion, decreased fertility and mortality | *Primary*: farmer questionnaires | Cattle, buffalo;  Animal;  Retrospective;  Not described |
| Benefit-cost analysis of foot-and-mouth disease vaccination at the farm-level in South Vietnam ^60^ | Vietnam, 2018 | "analyse the FMD financial impact at the farm-level in Vietnam and the BCR of the vaccination program" | *Economic evaluation of disease control:* stochastic partial budget model evaluating vaccination. Benefit cost ratio, net present value calculated. Selected sensitivity analysis. | Partial budget analysis: Additional costs: cost of vaccine and labour associated with vaccination; Saved costs: emergency vaccination, replacement costs/mortality, artificial insemination costs, treatment costs; Additional revenue: increased milk yield, selling healthy cattle, additional cattle raised and sold as lower mortality and abortion; Revenue forgone: income from dead/sick animals. Used to calculate benefit cost ratio, net present value. Sensitivity analysis for vaccination cost, price of cattle and price of milk. | *Primary*: farmer questionnaires, focus group discussions, semi-structured interviews  *Secondary*: previously published data | Cattle;  Household;  Prospective;  1 year |
| The exploration of financial impacts among three stakeholders during the 2016 foot-and-mouth disease outbreak in a dairy cooperative, Chiang Mai province, Thailand ^61^ | Thailand, 2019 | "Estimate the economic impact of FMD outbreaks in a dairy cooperative" | *Economic impact of disease:* deterministic summation of selected costs associated with direct and indirect impact of disease | Cost incurred due to disease: milk losses, treatment, and other changes in revenue at farm level; income at cooperative level and laboratory costs and logistic costs of disease control at provincial office | *Primary*: farmer questionnaires *Secondary*: official records | Dairy cattle;  Household, local, regional;  Retrospective;  June-September for 2 years |
| Foot and mouth disease outbreak investigation and estimation of its economic impact in selected districts in northwest Ethiopia ^62^ | Ethiopia, 2020 | "estimate economic impact of FMD outbreaks that occurred in 2017–2018 in selected areas in northwest Ethiopia" | *Economic impact of disease:* deterministic summation of selected costs associated with direct impact of disease | Cost incurred due to disease: reduction in milk yield, draught power loss, mortality loss, treatment cost | *Primary*: farmer questionnaire, market observation | Cattle;  Animal, household, local;  Retrospective;  1 outbreak |
| Economic impact assessment of foot-and-mouth disease burden and control in pastoral local dairy cattle production systems in Northern Nigeria: A cross-sectional survey ^63^ | Nigeria, 2020 | "assess economic impact of FMD in pastoral dairy cattle herds in Nigeria, and determine returns to investments in its control" | *Economic evaluation of disease control:* deterministic calculation of benefit cost ratio and net benefits using selected direct and indirect costs of disease. Selected sensitivity analysis | Cost incurred due to disease: production losses (morbidity: milk yield reduction, weight loss and mortality: died or culled). Disease control costs: antimicrobial treatment. Benefit cost ratio and net benefits calculated from these figures | *Primary*: qualitative questionnaire | Cattle;  Household, regional;  Prospective;  5 year |
| Socio-economic impact of Foot-and-Mouth Disease outbreaks and control measures: An analysis of Mongolian outbreaks in 2017 ^64^ | Mongolia, 2020 | "impact of FMD in Mongolia in 2017 by (a) assessing the socio-economic impact of FMD and the control measures on herders; and (b) estimating the national gross economic losses during 2017" | *Economic impact of disease:* deterministic summation of selected costs associated with direct and indirect impact of disease | Cost incurred due to disease: at farm level; impact on livelihood assets, impact on income due to forgone sales, impact on expenditure; other livelihood strategies. At national level; costs due to reaction expenditure (vaccination, diagnosis, outbreak investigation, compensation for culls, quarantine implementation) and production losses (mortality and compensation for culled animals) | *Primary*: farmer questionnaire *Secondary*: government data | Cattle, Sheep, Goats, Camels;  Farm, national;  Retrospective;  1 year |

1. Astudillo, V. M. & Auge de Mello, P. Cost and effectiveness analysis of two foot-and-mouth disease vaccination procedures. *Bol. del Cent. Panam. Fiebre Aft.* 49–63 (1980).

2. Crusius, V. A. & Lora, J. Q. Cost of vaccinating against foot and mouth disease in Peru. *Bol. del Cent. Panam. Fiebre Aft.* 21–25 (1981).

3. Munyua, S. J. M., Nguhiu-Mwangi, J., Njenga, J. & Karioki, D. I. An outbreak of Foot and mouth disease, and its socio-economic effects, in a herd of pigs in Nairobi, Kenya. *Bull. Anim. Heal. Prod. Africa* **39**, 51–55 (1991).

4. Riaz, M., Khan, M. S., Khan, M. A., Rabbani, A. & Arshad, M. Investigation on epidemiology and economic losses of major livestock diseases in District Gujarat. *Pak. Vet. J.* **12**, 86–88 (1992).

5. Ahuja, K. L., Prasad, S. & Kumar, A. Intensive surveillance of foot-and-mouth disease in selected livestock populations in Haryana. *Indian J. Virol.* **9**, 125–132 (1993).

6. Saxena, R. Economic value of some non-milk losses caused by foot-and-mouth disease (FMD) in India. *Working Paper - Institute of Rural Management (Anand)* 15 pp. (1994).

7. Ham, M. van, Zur, Y., Van Ham, M. & Zur, Y. Estimated damage to the Israeli dairy herd caused by foot and mouth disease outbreaks and a cost/benefit analysis of the present vaccination policy. *Isr. J. Vet. Med.* **49**, 13–16 (1994).

8. Saxena, R. Economic value of milk loss caused by foot-and-mouth disease (FMD) in India. *Working Paper - Institute of Rural Management (Anand)* 20 pp. (1994).

9. Tshering, P. An economic evaluation of the inpact of foot and mouth disease and its control in Bhutan. (University of Reading, 1995).

10. Singh, R., Tiwari, C. B. & Kumar, D. Statistical model for estimating losses due to foot-and-mouth disease in India. *Indian J. Anim. Sci.* **67**, 441–444 (1997).

11. Farag, M. A., Al-Sukayran, A., Mazloum, K. S. & Al-Bokmy, A. M. The role of small ruminants in the epizootiology of foot and mouth disease in Saudi Arabia with reference to the economic impact of the disease on sheep and goats. *Assiut Vet. Med. J.* **40**, 23–41 (1998).

12. Perry, B. D. *et al.* The economic impact of foot and mouth disease and its control in South-East Asia: a preliminary assessment with special reference to Thailand. *Rev. Sci. Tech. l’OIE* **18**, 478–497 (1999).

13. Randolph, T. F. *et al.* The economic impact of foot and mouth disease control and eradication in the Philippines. *OIE Rev. Sci. Tech.* **21**, 645–661 (2002).

14. Perry, B. D. *et al.* *The impact and poverty reduction implications of foot and mouth disease control in southern Africa*. (2003).

15. Suhail, S. M., Daur, U. A., Syed, M., Ahmed, N. & Ijaz, A. Prevalence of major livestock diseases in North Waziristan Agency. *Sarhad J. Agric.* **19**, 423–428 (2003).

16. Kimani, T. M., Mwirigi, J. W. & Murithi, R. M. Financial Impact Assessment of Foot and Mouth Disease in Large Scale Farms in Nakuru District, Kenya. *Kenya Vet.* **29**, 7–9 (2005).

17. Randolph, T. F., Morrison, J. A. & Poulton, C. Evaluating equity impacts of animal disease control the case of foot and mouth disease in Zimbabwe. *Rev. Agric. Econ.* **27**, 465–472 (2005).

18. Thirunavukkarasu, M. & Kathiravan, G. Economic losses due to mortality and culling of foot and mouth disease affected livestock. *Tamilnadu J. Vet. Anim. Sci.* **2**, 13–17 (2006).

19. Thirunavukkarasu, M. & Kathiravan, G. Monetary losses due to reproductive failures in FMD affected bovines. *Indian J. Dairy Sci.* **60**, 364–368 (2007).

20. Barasa, M. *et al.* Foot-and-mouth disease vaccination in South Sudan: Benefit-cost analysis and livelihoods impact. *Transbound. Emerg. Dis.* **55**, 339–351 (2008).

21. Mathew, L. & Menon, D. G. Economic impact of FMD in Chazhoor Panchayath. *Vet. World* **1**, 5–6 (2008).

22. Singh, B. & Prasad, S. Modelling of Economic Losses due to Some Important Diseases in Goats in India. *Agric. Econ. Res. Rev.* **21**, 297–302 (2008).

23. Șentürk, B. *et al.* Production losses due to endemic foot-and-mouth disease in cattle in Turkey. *Turkish J. Vet. Anim. Sci.* **32**, 433–440 (2008).

24. Rushton, J. Economic aspects of foot and mouth disease in Bolivia. *OIE Revue Scientifique et Technique* vol. 27 759–769 (2008).

25. Singh, B. & Prasad, S. Economic evaluation of important cattle diseases in India. *Indian Vet. J.* **85**, 1207–1210 (2008).

26. Singh, B. & Prasad, S. A model based assessment of economic losses due to some important diseases in sheep in India. *Indian J. Anim. Sci.* **79**, 1265–1268 (2009).

27. Rich, K. M., Perry, B. D. & Kaitibie, S. Commodity-based trade and market access for developing country livestock products: The case of beef exports from Ethiopia. *Int. Food Agribus. Manag. Rev.* **12**, 1–22 (2009).

28. Rast, L., Windsor, P. A. & Khounsy, S. Limiting the impacts of foot and mouth disease in large ruminants in northern Lao People’s Democratic Republic by vaccination: a case study. *Transbound. Emerg. Dis.* **57**, 147–153 (2010).

29. Roy Chowdhury, S. *et al.* Simulative modeling to control the Foot and Mouth Disease epidemic. *Procedia Comput. Sci.* **1**, 2261–2270 (2010).

30. El-Hussein, A. M. & Daboura, A. Economic impact of an outbreak of foot and mouth disease in Khartoum State, Sudan. *Vet. World* **4**, 219–222 (2012).

31. Shankar, B., Morzaria, S., Fiorucci, A. & Hak, M. Animal disease and livestock-keeper livelihoods in Southern Cambodia. *Int. Dev. Plan. Rev.* **34**, 39–63 (2012).

32. Ashenafi, B. Costs and benefits of foot and mouth disease vaccination in commercial dairy farms in Central Ethiopia. (Wageningen University, 2012). doi:10.13140/RG.2.2.29650.35529.

33. Young, J. R., Suon, S., Andrews, C. J., Henry, L. A. & Windsor, P. A. Assessment of Financial Impact of Foot and Mouth Disease on Smallholder Cattle Farmers in Southern Cambodia. *Transbound. Emerg. Dis.* **60**, 166–174 (2013).

34. Singh, B., Prasad, S., Sinha, D. K. & Verma, M. E. D. R. Estimation of economic losses due to foot and mouth disease in India. *Indian J. Anim. Sci.* **83**, 964–970 (2013).

35. Paul, D., Chandel, B. S. & Ray, J. Quantity and value of milk losses due to technical constraints - a case of crossbred cows in north-eastern states of India. *Indian J. Agric. Econ.* **68**, 562–572 (2013).

36. Jemberu, W. T., Mourits, M. C. M. M., Woldehanna, T. & Hogeveen, H. Economic impact of foot and mouth disease outbreaks on smallholder farmers in Ethiopia. *Prev. Vet. Med.* **116**, 26–36 (2014).

37. Gezahegn, A. *et al.* Seroprevalence of foot and mouth disease (FMD) and associated economic impact on Central Ethiopian cattle feedlots. *J. Vet. Med. Anim. Heal.* **6**, 154–158 (2014).

38. Knight-Jones, T. J. D., Njeumi, F., Elsawalhy, A., Wabacha, J. & Rushton, J. Risk assessment and cost-effectiveness of animal health certification methods for livestock export in Somalia. *Prev. Vet. Med.* **113**, 469–483 (2014).

39. Birhanu, T. Prevalence of the major infectious animal diseases affecting livestock trade industry in Ethiopia. *J. Biol. Agric. Healthc.* **4**, 76–82 (2014).

40. Baluka, S. A., Ocaido, M. & Mugisha, A. Prevalence and economic importance of Foot and Mouth Disease, and Contagious Bovine Pleuropneumonia Outbreaks in cattle in Isingiro and Nakasongola Districts of Uganda. *Discourse J. Agric. Food Sci.* **2**, 107–117 (2014).

41. Baluka, S. A., Mugisha, A. & Ocaido, M. Financial impact of Foot and Mouth Disease and Contagious Bovine Pleuropneumonia along the cattle marketing chain in selected districts in Uganda. *Livest. Res. Rural Dev.* **26**, Article 170 (2014).

42. Ferrari, G., Tasciotti, L., Khan, E. & Kiani, A. Foot-and-mouth disease and its effect on milk yield: an economic analysis on livestock holders in Pakistan. *Transbound. Emerg. Dis.* **61**, e52-9 (2014).

43. Abao, L. N. B., Kono, H., Gunarathne, A., Promentilla, R. R. & Gaerlan, M. Z. Impact of foot-and-mouth disease on pork and chicken prices in Central Luzon, Philippines. *Prev. Vet. Med.* **113**, 398–406 (2014).

44. Centre for Development Oriented Research in Agriculture and Livelihood Systems. *Assessment of socio-economic impacts of Foot-and-Mouth Disease on Cattle in Twelve Villages of Cambodia*. https://doc.oie.int/dyn/portal/index.seam?page=alo&aloId=35149 (2014) doi:https://doi.org/10.20506/standz.2779.

45. Naziri, D., Rich, K. M. & Bennett, B. Would a Commodity-based Trade Approach Improve Market Access for Africa? A Case Study of the Potential of Beef Exports from Communal Areas of Namibia. *Dev. POLICY Rev.* **33**, 195–219 (2015).

46. Ashfaq, M., Razzaq, A., Shamsheer-ul-Haq & Muhammad, G. Economic analysis of dairy animal diseases in Punjab: a case study of Faisalabad district. *J. Anim. PLANT Sci.* **25**, 1482–1495 (2015).

47. Nampanya, S. *et al.* Financial Impact of Foot and Mouth Disease on Large Ruminant Smallholder Farmers in the Greater Mekong Subregion. *Transbound. Emerg. Dis.* **62**, 555–564 (2015).

48. Roy, R., Tiwari, R. & Dutt, T. Incidence of important goat diseases and economic losses under field condition. *Indian J. Anim. Sci.* **85**, 24–26 (2015).

49. Baluka, S. A. Economic effects of foot and mouth disease outbreaks along the cattle marketing chain in Uganda. *Vet. WORLD* **9**, 544–553 (2016).

50. Jemberu, W. T., Mourits, M., Rushton, J. & Hogeveen, H. Cost-benefit analysis of foot and mouth disease control in Ethiopia. *Prev. Vet. Med.* **132**, 67–82 (2016).

51. Young, J. R. *et al.* Benefit-Cost Analysis of Foot and Mouth Disease Control in Large Ruminants in Cambodia. *Transbound. Emerg. Dis.* **63**, 508–522 (2016).

52. Nampanya, S. *et al.* Financial Impacts of Foot-and-Mouth Disease at Village and National Levels in Lao PDR. *Transbound. Emerg. Dis.* **63**, E403–E411 (2016).

53. Nampanya, S., Khounsy, S., Abila, R., Dy, C. & Windsor, P. A. Household Financial Status and Gender Perspectives in Determining the Financial Impact of Foot and Mouth Disease in Lao PDR. *Transbound. Emerg. Dis.* **63**, 398–407 (2016).

54. Farooq, U. *et al.* Epidemiological analysis and economic impact assessment of foot-and-mouth disease at Landhi Dairy Colony Karachi. *Asian J. Agric. Biol.* **5**, 7–14 (2017).

55. Govindaraj, G. *et al.* Farm Community Impacts of Foot-and-Mouth Disease Outbreaks in Cattle and Buffaloes in Karnataka State, India. *Transbound. Emerg. Dis.* **64**, 849–860 (2017).

56. Pham, H. T. T. *et al.* Financial Impacts of Priority Swine Diseases to Pig Farmers in Red River and Mekong River Delta, Vietnam. *Transbound. Emerg. Dis.* **64**, 1168–1177 (2017).

57. Casey-Bryars, M. *et al.* Waves of endemic foot-and-mouth disease in eastern Africa suggest feasibility of proactive vaccination approaches. *Nat. Ecol. Evol.* **2**, 1449–1457 (2018).

58. Souley Kouato, B. *et al.* Spatio-temporal patterns of foot-and-mouth disease transmission in cattle between 2007 and 2015 and quantitative assessment of the economic impact of the disease in Niger. *Transbound. Emerg. Dis.* **65**, 1049–1066 (2018).

59. Sinha, M. K., Thombare, N. N., Mondal, B., Meena, M. S. & Kumar, P. Analysis of foot and mouth disease in dairy animals: An assessment of cost and loss from sample farmers. *Indian J. Anim. Res.* **52**, 754–757 (2018).

60. Dinh Bao, T. *et al.* Benefit-cost analysis of foot-and-mouth disease vaccination at the farm-level in South Vietnam. *Front. Vet. Sci.* **5**, 26 (2018).

61. Jongthanachote, W., Poontrakulkeat, M., Mungthisarn, K., Wiratsudakul, A. & Jiemtaweeboon, S. The exploration of financial impacts among three stakeholders during the 2016 foot-and-mouth disease outbreak in a dairy cooperative, Chiang Mai province, Thailand. *J. Appl. Anim. Sci.* **12**, 31–40 (2019).

62. Tadesse, B., Tesfahun, A., Molla, W., Demisse, E. & Jemberu, W. T. Foot and mouth disease outbreak investigation and estimation of its economic impact in selected districts in northwest Ethiopia. *Vet. Med. Sci.* **6**, 122–132 (2020).

63. Alhaji, N. B. *et al.* Economic impact assessment of foot-and-mouth disease burden and control in pastoral local dairy cattle production systems in Northern Nigeria: A cross-sectional survey. *Prev. Vet. Med.* **177**, 104974 (2020).

64. Limon, G. *et al.* Socio-economic impact of Foot-and-Mouth Disease outbreaks and control measures: An analysis of Mongolian outbreaks in 2017. *Transbound. Emerg. Dis.* **67**, 2034–2049 (2020).
