# Supplementary material for: A systematic review of the methods used to analyze the economic impact of endemic foot‐and‐mouth disease
Source: Transbound Emerg Dis. 2022 Jun 20;69(5):e2249–60. doi: 10.1111/tbed.14564 (PMC9795869; doi:10.1111/tbed.14564)
Supplement: Supplementary file 2 — List S2 [file TBED-69-e2249-s001.docx]

1. Astudillo, V. M. & Auge de Mello, P. Cost and effectiveness analysis of two foot-and-mouth disease vaccination procedures. *Bol. del Cent. Panam. Fiebre Aft.* 49–63 (1980).

2. Crusius, V. A. & Lora, J. Q. Cost of vaccinating against foot and mouth disease in Peru. *Bol. del Cent. Panam. Fiebre Aft.* 21–25 (1981).

3. Munyua, S. J. M., Nguhiu-Mwangi, J., Njenga, J. & Karioki, D. I. An outbreak of Foot and mouth disease, and its socio-economic effects, in a herd of pigs in Nairobi, Kenya. *Bull. Anim. Heal. Prod. Africa* **39**, 51–55 (1991).

4. Riaz, M., Khan, M. S., Khan, M. A., Rabbani, A. & Arshad, M. Investigation on epidemiology and economic losses of major livestock diseases in District Gujarat. *Pak. Vet. J.* **12**, 86–88 (1992).

5. Ahuja, K. L., Prasad, S. & Kumar, A. Intensive surveillance of foot-and-mouth disease in selected livestock populations in Haryana. *Indian J. Virol.* **9**, 125–132 (1993).

6. Saxena, R. Economic value of some non-milk losses caused by foot-and-mouth disease (FMD) in India. *Working Paper - Institute of Rural Management (Anand)* 15 pp. (1994).

7. Ham, M. van, Zur, Y., Van Ham, M. & Zur, Y. Estimated damage to the Israeli dairy herd caused by foot and mouth disease outbreaks and a cost/benefit analysis of the present vaccination policy. *Isr. J. Vet. Med.* **49**, 13–16 (1994).

8. Saxena, R. Economic value of milk loss caused by foot-and-mouth disease (FMD) in India. *Working Paper - Institute of Rural Management (Anand)* 20 pp. (1994).

9. Tshering, P. An economic evaluation of the inpact of foot and mouth disease and its control in Bhutan. (University of Reading, 1995).

10. Singh, R., Tiwari, C. B. & Kumar, D. Statistical model for estimating losses due to foot-and-mouth disease in India. *Indian J. Anim. Sci.* **67**, 441–444 (1997).

11. Farag, M. A., Al-Sukayran, A., Mazloum, K. S. & Al-Bokmy, A. M. The role of small ruminants in the epizootiology of foot and mouth disease in Saudi Arabia with reference to the economic impact of the disease on sheep and goats. *Assiut Vet. Med. J.* **40**, 23–41 (1998).

12. Perry, B. D. *et al.* The economic impact of foot and mouth disease and its control in South-East Asia: a preliminary assessment with special reference to Thailand. *Rev. Sci. Tech. l’OIE* **18**, 478–497 (1999).

13. Randolph, T. F. *et al.* The economic impact of foot and mouth disease control and eradication in the Philippines. *OIE Rev. Sci. Tech.* **21**, 645–661 (2002).

14. Perry, B. D. *et al.* *The impact and poverty reduction implications of foot and mouth disease control in southern Africa*. (2003).

15. Suhail, S. M., Daur, U. A., Syed, M., Ahmed, N. & Ijaz, A. Prevalence of major livestock diseases in North Waziristan Agency. *Sarhad J. Agric.* **19**, 423–428 (2003).

16. Kimani, T. M., Mwirigi, J. W. & Murithi, R. M. Financial Impact Assessment of Foot and Mouth Disease in Large Scale Farms in Nakuru District, Kenya. *Kenya Vet.* **29**, 7–9 (2005).

17. Randolph, T. F., Morrison, J. A. & Poulton, C. Evaluating equity impacts of animal disease control the case of foot and mouth disease in Zimbabwe. *Rev. Agric. Econ.* **27**, 465–472 (2005).

18. Thirunavukkarasu, M. & Kathiravan, G. Economic losses due to mortality and culling of foot and mouth disease affected livestock. *Tamilnadu J. Vet. Anim. Sci.* **2**, 13–17 (2006).

19. Thirunavukkarasu, M. & Kathiravan, G. Monetary losses due to reproductive failures in FMD affected bovines. *Indian J. Dairy Sci.* **60**, 364–368 (2007).

20. Barasa, M. *et al.* Foot-and-mouth disease vaccination in South Sudan: Benefit-cost analysis and livelihoods impact. *Transbound. Emerg. Dis.* **55**, 339–351 (2008).

21. Mathew, L. & Menon, D. G. Economic impact of FMD in Chazhoor Panchayath. *Vet. World* **1**, 5–6 (2008).

22. Singh, B. & Prasad, S. Modelling of Economic Losses due to Some Important Diseases in Goats in India. *Agric. Econ. Res. Rev.* **21**, 297–302 (2008).

23. Șentürk, B. *et al.* Production losses due to endemic foot-and-mouth disease in cattle in Turkey. *Turkish J. Vet. Anim. Sci.* **32**, 433–440 (2008).

24. Rushton, J. Economic aspects of foot and mouth disease in Bolivia. *OIE Revue Scientifique et Technique* vol. 27 759–769 (2008).

25. Singh, B. & Prasad, S. Economic evaluation of important cattle diseases in India. *Indian Vet. J.* **85**, 1207–1210 (2008).

26. Singh, B. & Prasad, S. A model based assessment of economic losses due to some important diseases in sheep in India. *Indian J. Anim. Sci.* **79**, 1265–1268 (2009).

27. Rich, K. M., Perry, B. D. & Kaitibie, S. Commodity-based trade and market access for developing country livestock products: The case of beef exports from Ethiopia. *Int. Food Agribus. Manag. Rev.* **12**, 1–22 (2009).

28. Rast, L., Windsor, P. A. & Khounsy, S. Limiting the impacts of foot and mouth disease in large ruminants in northern Lao People’s Democratic Republic by vaccination: a case study. *Transbound. Emerg. Dis.* **57**, 147–153 (2010).

29. Roy Chowdhury, S. *et al.* Simulative modeling to control the Foot and Mouth Disease epidemic. *Procedia Comput. Sci.* **1**, 2261–2270 (2010).

30. El-Hussein, A. M. & Daboura, A. Economic impact of an outbreak of foot and mouth disease in Khartoum State, Sudan. *Vet. World* **4**, 219–222 (2012).

31. Shankar, B., Morzaria, S., Fiorucci, A. & Hak, M. Animal disease and livestock-keeper livelihoods in Southern Cambodia. *Int. Dev. Plan. Rev.* **34**, 39–63 (2012).

32. Ashenafi, B. Costs and benefits of foot and mouth disease vaccination in commercial dairy farms in Central Ethiopia. (Wageningen University, 2012). doi:10.13140/RG.2.2.29650.35529.

33. Young, J. R., Suon, S., Andrews, C. J., Henry, L. A. & Windsor, P. A. Assessment of Financial Impact of Foot and Mouth Disease on Smallholder Cattle Farmers in Southern Cambodia. *Transbound. Emerg. Dis.* **60**, 166–174 (2013).

34. Singh, B., Prasad, S., Sinha, D. K. & Verma, M. E. D. R. Estimation of economic losses due to foot and mouth disease in India. *Indian J. Anim. Sci.* **83**, 964–970 (2013).

35. Paul, D., Chandel, B. S. & Ray, J. Quantity and value of milk losses due to technical constraints - a case of crossbred cows in north-eastern states of India. *Indian J. Agric. Econ.* **68**, 562–572 (2013).

36. Jemberu, W. T., Mourits, M. C. M. M., Woldehanna, T. & Hogeveen, H. Economic impact of foot and mouth disease outbreaks on smallholder farmers in Ethiopia. *Prev. Vet. Med.* **116**, 26–36 (2014).

37. Gezahegn, A. *et al.* Seroprevalence of foot and mouth disease (FMD) and associated economic impact on Central Ethiopian cattle feedlots. *J. Vet. Med. Anim. Heal.* **6**, 154–158 (2014).

38. Knight-Jones, T. J. D., Njeumi, F., Elsawalhy, A., Wabacha, J. & Rushton, J. Risk assessment and cost-effectiveness of animal health certification methods for livestock export in Somalia. *Prev. Vet. Med.* **113**, 469–483 (2014).

39. Birhanu, T. Prevalence of the major infectious animal diseases affecting livestock trade industry in Ethiopia. *J. Biol. Agric. Healthc.* **4**, 76–82 (2014).

40. Baluka, S. A., Ocaido, M. & Mugisha, A. Prevalence and economic importance of Foot and Mouth Disease, and Contagious Bovine Pleuropneumonia Outbreaks in cattle in Isingiro and Nakasongola Districts of Uganda. *Discourse J. Agric. Food Sci.* **2**, 107–117 (2014).

41. Baluka, S. A., Mugisha, A. & Ocaido, M. Financial impact of Foot and Mouth Disease and Contagious Bovine Pleuropneumonia along the cattle marketing chain in selected districts in Uganda. *Livest. Res. Rural Dev.* **26**, Article 170 (2014).

42. Ferrari, G., Tasciotti, L., Khan, E. & Kiani, A. Foot-and-mouth disease and its effect on milk yield: an economic analysis on livestock holders in Pakistan. *Transbound. Emerg. Dis.* **61**, e52-9 (2014).

43. Abao, L. N. B., Kono, H., Gunarathne, A., Promentilla, R. R. & Gaerlan, M. Z. Impact of foot-and-mouth disease on pork and chicken prices in Central Luzon, Philippines. *Prev. Vet. Med.* **113**, 398–406 (2014).

44. Centre for Development Oriented Research in Agriculture and Livelihood Systems. *Assessment of socio-economic impacts of Foot-and-Mouth Disease on Cattle in Twelve Villages of Cambodia*. https://doc.oie.int/dyn/portal/index.seam?page=alo&aloId=35149 (2014) doi:https://doi.org/10.20506/standz.2779.

45. Naziri, D., Rich, K. M. & Bennett, B. Would a Commodity-based Trade Approach Improve Market Access for Africa? A Case Study of the Potential of Beef Exports from Communal Areas of Namibia. *Dev. POLICY Rev.* **33**, 195–219 (2015).

46. Ashfaq, M., Razzaq, A., Shamsheer-ul-Haq & Muhammad, G. Economic analysis of dairy animal diseases in Punjab: a case study of Faisalabad district. *J. Anim. PLANT Sci.* **25**, 1482–1495 (2015).

47. Nampanya, S. *et al.* Financial Impact of Foot and Mouth Disease on Large Ruminant Smallholder Farmers in the Greater Mekong Subregion. *Transbound. Emerg. Dis.* **62**, 555–564 (2015).

48. Roy, R., Tiwari, R. & Dutt, T. Incidence of important goat diseases and economic losses under field condition. *Indian J. Anim. Sci.* **85**, 24–26 (2015).

49. Baluka, S. A. Economic effects of foot and mouth disease outbreaks along the cattle marketing chain in Uganda. *Vet. WORLD* **9**, 544–553 (2016).

50. Jemberu, W. T., Mourits, M., Rushton, J. & Hogeveen, H. Cost-benefit analysis of foot and mouth disease control in Ethiopia. *Prev. Vet. Med.* **132**, 67–82 (2016).

51. Young, J. R. *et al.* Benefit-Cost Analysis of Foot and Mouth Disease Control in Large Ruminants in Cambodia. *Transbound. Emerg. Dis.* **63**, 508–522 (2016).

52. Nampanya, S. *et al.* Financial Impacts of Foot-and-Mouth Disease at Village and National Levels in Lao PDR. *Transbound. Emerg. Dis.* **63**, E403–E411 (2016).

53. Nampanya, S., Khounsy, S., Abila, R., Dy, C. & Windsor, P. A. Household Financial Status and Gender Perspectives in Determining the Financial Impact of Foot and Mouth Disease in Lao PDR. *Transbound. Emerg. Dis.* **63**, 398–407 (2016).

54. Farooq, U. *et al.* Epidemiological analysis and economic impact assessment of foot-and-mouth disease at Landhi Dairy Colony Karachi. *Asian J. Agric. Biol.* **5**, 7–14 (2017).

55. Govindaraj, G. *et al.* Farm Community Impacts of Foot-and-Mouth Disease Outbreaks in Cattle and Buffaloes in Karnataka State, India. *Transbound. Emerg. Dis.* **64**, 849–860 (2017).

56. Pham, H. T. T. *et al.* Financial Impacts of Priority Swine Diseases to Pig Farmers in Red River and Mekong River Delta, Vietnam. *Transbound. Emerg. Dis.* **64**, 1168–1177 (2017).

57. Casey-Bryars, M. *et al.* Waves of endemic foot-and-mouth disease in eastern Africa suggest feasibility of proactive vaccination approaches. *Nat. Ecol. Evol.* **2**, 1449–1457 (2018).

58. Souley Kouato, B. *et al.* Spatio-temporal patterns of foot-and-mouth disease transmission in cattle between 2007 and 2015 and quantitative assessment of the economic impact of the disease in Niger. *Transbound. Emerg. Dis.* **65**, 1049–1066 (2018).

59. Sinha, M. K., Thombare, N. N., Mondal, B., Meena, M. S. & Kumar, P. Analysis of foot and mouth disease in dairy animals: An assessment of cost and loss from sample farmers. *Indian J. Anim. Res.* **52**, 754–757 (2018).

60. Dinh Bao, T. *et al.* Benefit-cost analysis of foot-and-mouth disease vaccination at the farm-level in South Vietnam. *Front. Vet. Sci.* **5**, 26 (2018).

61. Jongthanachote, W., Poontrakulkeat, M., Mungthisarn, K., Wiratsudakul, A. & Jiemtaweeboon, S. The exploration of financial impacts among three stakeholders during the 2016 foot-and-mouth disease outbreak in a dairy cooperative, Chiang Mai province, Thailand. *J. Appl. Anim. Sci.* **12**, 31–40 (2019).

62. Tadesse, B., Tesfahun, A., Molla, W., Demisse, E. & Jemberu, W. T. Foot and mouth disease outbreak investigation and estimation of its economic impact in selected districts in northwest Ethiopia. *Vet. Med. Sci.* **6**, 122–132 (2020).

63. Alhaji, N. B. *et al.* Economic impact assessment of foot-and-mouth disease burden and control in pastoral local dairy cattle production systems in Northern Nigeria: A cross-sectional survey. *Prev. Vet. Med.* **177**, 104974 (2020).

64. Limon, G. *et al.* Socio-economic impact of Foot-and-Mouth Disease outbreaks and control measures: An analysis of Mongolian outbreaks in 2017. *Transbound. Emerg. Dis.* **67**, 2034–2049 (2020).
