# Supplementary material for: A systematic review of the methods used to analyze the economic impact of endemic foot‐and‐mouth disease
Source: Transbound Emerg Dis. 2022 Jun 20;69(5):e2249–60. doi: 10.1111/tbed.14564 (PMC9795869; doi:10.1111/tbed.14564)
Supplement: Supplementary file 3 — Figure S1 [file TBED-69-e2249-s003.docx]

|  |
| --- |
| **Figure S1.** Post hoc analysis of influence of denominator in quality score using Pearson’s Correlation Coefficient. |
